# Protective factors for adolescent sexual risk behaviours and experiences linked to HIV infection in South Africa: a three-wave longitudinal analysis of caregiving, education, food security, and social protection

**DOI:** 10.1186/s12889-023-16373-5

**Published:** 2023-07-29

**Authors:** William E. Rudgard, Maria Granvik Saminathen, Mark Orkin, Boladé Hamed Banougnin, Yulia Shenderovich, Elona Toska

**Affiliations:** 1 Department of Social Policy and Intervention, University of Oxford, Oxford, OX1 2ER UK; 2 Centre for Social Science Research, University of Cape Town, Cape Town, South Africa; 3 MRC/Wits Development Pathways to Health Research Unit, School of Clinical Medicine, University of the Witwatersrand, Johannesburg, South Africa; 4 Wolfson Centre for Young People’s Mental Health, Cardiff University, Cardiff, UK; 5 Centre for the Development and Evaluation of Complex Interventions for Public Health Improvement (DECIPHer), School of Social Sciences, Cardiff University, Cardiff, UK; 6 Department of Sociology, University of Cape Town, Cape Town, South Africa

**Keywords:** Adolescent, HIV, Prevention, Condomless sex, Transactional sex, Structural factors, Education, Parenting, South Africa

## Abstract

**Background:**

Structural interventions are endorsed to enhance biomedical and behavioural HIV prevention programmes for adolescents. Aiming to inform future interventions, we evaluated longitudinal associations between six protective factors that link closely to existing structural HIV prevention interventions, and five sexual risk behaviours for HIV transmission in a cohort of adolescents in South Africa.

**Methods:**

We used three rounds of data between 2014–2018 on 1046 adolescents living with HIV and 473 age-matched community peers in South Africa’s Eastern Cape (Observations = 4402). We estimated sex-specific associations between six time-varying protective factors − number of social grants, education enrolment, days with enough food, caregiver supervision, positive caregiving, and adolescent-caregiver communication; and five HIV risk behaviours − multiple sexual partners, transactional sex, age-disparate sex, condomless sex, and sex on substances. HIV risk behaviours were analysed separately in multivariable random effects within-between logistic regression models that accounted for correlation of repeated observations on the same individual. We calculated prevalence ratios (PR), contrasting adjusted probabilities of HIV risk behaviours at ‘No’ and ‘Yes’ for education enrolment, and average and maximum values for the other five protective factors.

**Results:**

The sample mean age was 15.29 (SD: 3.23) years and 58% were girls. Among girls, within-individuals, increases from mean to maximum scores in positive caregiving were associated with lower probability of transactional sex (PR = 0.79; 95%CI = 0.67–0.91); in caregiver supervision were associated with lower probability of transactional sex (PR = 0.75; 95%CI = 0.66–0.84), and age-disparate sex (PR = 0.84; 95%CI = 0.73–0.95); in adolescent-caregiver communication were associated with higher probability of transactional sex (PR = 1.70; 95%CI = 1.08–2.32); and in days with enough food at home were associated with lower probability of multiple sexual partners (PR = 0.89; 95%CI = 0.81–0.97), and transactional sex (PR = 0.82; 95%CI = 0.72–0.92). Change from non-enrolment in education to enrolment was associated with lower probability of age-disparate sex (PR = 0.49; 95%CI = 0.26–0.73). Between-individuals, relative to mean caregiver supervision scores, maximum scores were associated with lower probability of multiple sexual partners (PR = 0.59; 95%CI = 0.46–0.72), condomless sex (PR = 0.80; 95%CI = 0.69–0.91), and sex on substances (PR = 0.42; 95%CI = 0.26–0.59); and relative to non-enrolment, education enrolment was associated with lower probability of condomless sex (PR = 0.59; 95%CI = 0.39–0.78). Among boys, within-individuals, increases from mean to maximum scores in positive caregiving were associated with lower probability of transactional sex (PR = 0.77; 95%CI = 0.59–0.96), and higher probability of condomless sex (PR = 1.26; 95%CI = 1.08–1.43); in caregiver supervision were associated with lower probability of multiple sexual partners (PR = 0.73; 95%CI = 0.64–0.82), transactional sex (PR = 0.63; 95%CI = 0.50–0.76), age-disparate sex (PR = 0.67; 95%CI = 0.49–0.85), and sex on substances (PR = 0.61; 95%CI = 0.45–0.78), and in days with enough food at home were associated with lower probability of transactional sex (PR = 0.91; 95%CI = 0.84–0.98).

**Conclusion:**

Effective structural interventions to improve food security and education enrolment among adolescent girls, and positive and supervisory caregiving among adolescent girls and boys are likely to translate into crucial reductions in sexual risk behaviours linked to HIV transmission in this population.

**Supplementary Information:**

The online version contains supplementary material available at 10.1186/s12889-023-16373-5.

## Background

In Eastern and Southern Africa, rates of new HIV infections remain well above the Joint United Nations Programme on HIV/AIDS (UNAIDS) targets for ending AIDS as a public health threat by 2030 [1–3]. Adolescents, and especially adolescent girls, have been identified as a group at disproportionate risk of HIV infection. In 2022, 37% of all new HIV infections in the region were among adolescents, with adolescent girls accounting for 84% of them [4, 5].

Sexual risk behaviours and experiences including condomless sex, early sexual debut, multiple sexual partners, transactional sex, age-disparate sex, and sex on substances are significant drivers of HIV transmission among adolescents—compounded by low rates of HIV testing, HIV status disclosure to partners and antiretroviral therapy (ART) adherence in this group [6–19]. Consistent with contemporary theories in social epidemiology, adolescents’ engagement in these sexual risk behaviours is strongly linked to the broader social and economic conditions that they live and grow up in, including intimate partner violence, caregiver support, and access to basic needs such as education, housing, and food security [19–28]. Persistent gender inequalities and harmful norms mean that adolescent girls continue to experience unequal exposure to these conditions compared to adolescent boys [29–31].

To accelerate reductions in HIV incidence among adolescents, UNAIDS endorses a combination approach to HIV prevention that prioritises structural interventions alongside biomedical and behavioural interventions [32]. Using the UNAIDS definition, structural interventions ‘seek to alter the physical, legal and social environment in which individual behaviour takes place’ [33]. Examples of structural interventions for HIV prevention include anti-poverty cash transfers to increase household income and improve girls’ education attendance [34–38]; caregiver support programmes that reduce violence victimisation by caregivers and improve food security [39]; and gender transformative participatory approaches that reduce intimate partner violence perpetration [40, 41]. Multiple structural interventions can also be combined to address distinct social and economic conditions linked to HIV transmission simultaneously, for example cash transfers plus gender transformative participatory approaches [42–44]. The ‘DREAMS’ initiative set up by PEPFAR and partners in 2015 is an ambitious initiative to combine 12 evidence-based structural, biomedical, and behavioural interventions to strengthen HIV prevention amongst young women in sub-Saharan Africa [45].

Multiple high-quality studies support the potential of cash transfers and education subsidies as structural interventions to prevent new HIV infections and associated risk behaviours in adolescents [46–48]. There is also evidence that the ‘DREAMS’ initiative is likely to promote safer sexual partnerships when implemented sustainably [49]. However, the evidence-base for structural interventions remains mixed, with some evaluations finding no evidence of their effects on HIV incidence and risk behaviours [46, 47]. Reviews have highlighted that in some cases null effects may be driven by studies’ selective focus on school-based samples, which by their design exclude adolescents that are not enrolled in education and highly vulnerable to HIV infection [50, 51]. High-quality longitudinal studies of community-based samples can address this limitation and provide further insight into how changes in the social and economic conditions of adolescents’ lives are associated with HIV risk. Structural interventions targeting these conditions can then be prioritised for further evaluation.

To our knowledge, most observational studies linking socio-economic conditions to adolescent HIV risk have been cross-sectional or based on two-waves [51–53]. Compared to these designs, studies with three or more waves (multi-wave designs) have several important advantages for validity and reliability. They can more effectively distinguish true change from measurement error, inform the shape of individuals’ exposure or outcome trajectories, and investigate links between prior predictor status and outcomes – better controlling for reverse causality [54, 55]. Aiming to inform the design of future structural interventions for HIV prevention in adolescents, we used a multi-wave analysis to evaluate the longitudinal associations between six time-varying protective factors and five sexual risk behaviours linked to HIV transmission among adolescents.

## Methods

Our analysis used three waves of data from the Mzantsi Wakho (‘Our South Africa’) study in the Eastern Cape Province, South Africa, which was set up to identify risk and resilience-promoting factors for adolescent sexual and reproductive health, and adherence to long-term medication [56].

The Eastern Cape is one of the two poorest provinces in South Africa with 79% of children estimated to be multidimensionally poor (i.e. deprived in three or more out of seven dimensions of well-being), as compared to 62% nationally [57]. In 2019, 61% of households were recorded to have received at least one social grant, as compared to 46% nationally [58]. The prevalence of HIV is also higher than the national average with 12% of adolescents and young adults aged 15–24 years estimated to be living with HIV in 2017, as compared to 8% nationally [59].

We report the analysis according to the Strengthening the Reporting of Observational Studies in Epidemiology checklist, Supplementary Table 1, Additional File 1 [60].

### Study sample

Adolescents aged 10 to 19 living with HIV and not living with HIV participating in the Mzantsi Wakho study. Recruitment for Mzantsi Wakho took place between March 2014 and September 2015 in the Amathole District (AD) and Buffalo City Metropolitan (BCM) municipalities of the Eastern Cape. First, all adolescents who had ever initiated HIV care in one of the 53 health facilities providing ART to adolescents in AD and BCM municipalities were contacted and invited to participate in the study. Initiation of ART was ascertained from paper and electronic clinical files. Second, to avoid stigmatization of adolescents living with HIV during data collection, a sub-sample of cohabiting/neighbouring adolescents who had never initiated HIV care were also invited to participate in the study.

Ninety percent of eligible and invited participants were enrolled in the study at wave one. Survey follow-up rates at wave two [November 2015 and March 2017 (~ 17 months)] and wave three [April 2017 and March 2018 (~ 31 months)] were 93% and 91%, respectively. Retention strategies included a strong emphasis on building rapport between fieldworkers and participants, including over an 8-week pre-study community interaction period, and travel across South Africa to interview participants that had moved outside of the study location at follow-up. An additional 34 adolescents that had initiated HIV care but were not interviewed during wave 1 were recruited during wave 2, together with 10 additional community peers.

### Ethics

Ethical approvals were obtained from the University of Oxford [SSD/CUREC2/12–21; R43892/RE003], University of Cape Town [CSSR 2013/4; CSSR 2019/01], Provincial Departments of Health and Basic Education, health facilities and schools. Voluntary informed written and verbal consent was obtained from all respondents, and their parent/ guardians when they were under 18 years. No financial incentives were given for participation, but adolescents received a certificate and small gift pack with snacks and toiletries. Interviews took place in Xhosa or English, according to participant choice. Confidentiality was maintained except when participants disclosed serious risk of harm to themselves or others, in which case a healthcare worker was informed in line with Sect. 14 of the South African National Health Act [61]. Reports of recent abuse, rape or suicidality were immediately supported with access to counselling, post-exposure prophylaxis, pregnancy prevention, and child protection measures [62].

### Measures

Measures and scales were pre-piloted with a group of local adolescent advisors, and feedback on questionnaire design was given by the South African National Departments of Health, Basic Education, and Social Development, the South African National AIDS Council, UNICEF, PEPFAR, USAID and local non-governmental organisations. All questionnaires are available at www.youngcarers.org.za.

#### HIV risk behaviours

We assessed five risk behaviours for penile-vaginal sex with evidence linking them to HIV transmission among adolescents in sub-Saharan Africa. Questions were adapted from the National Survey of HIV and Risk Behaviour amongst young South Africans, the PREPARE trial and the Child Behavior Checklist Youth Self-Report [63, 64]. They were: (1) *Multiple sexual partners*, as having more than one sexual partner [9, 10]; (2) *Transactional sex,* as receipt of money, drinks, clothes, mobile airtime, a place to stay, lifts in a car/taxi, better marks at school, school fees, food, or other kinds of material exchange for having sex with someone [18, 19]; (3) *Age-disparate sex,* as having a sexual partner at least five years older [6, 11]; (4) *Condomless sex,* as not using a condom for the duration of sex at least once [8]; (5) *Sex on substances,* as having sex when drunk, smoking dagga, or using any other drugs [12]. All participants were asked about ever having sex, and whether they had had sex in the last 12 months. Those responding positively to both of these questions were asked about sexual risk behaviours with a recall period of 12 months, except for transactional sex at wave one, which was reported with a recall period of six months.

#### Hypothesised protective factors

We evaluated six modifiable protective factors hypothesised to be associated with lower odds of engaging in sexual risk behaviours and that link closely to existing structural HIV prevention interventions [45]. (1) *Number of social grants*, as the total number of South African Social Security Agency (SASSA) grants received by the adolescent and their household; (2) *Positive caregiving*, as a sum of the six items that make up the positive caregiving subscale of the Alabama Parenting Questionnaire scale, which considers warmth and praise from a primary caregiver (range: 0–24) [65]; (3) *Caregiver supervision*, as a sum of the reverse scores of 10 items that make up the monitoring & supervision subscale of the Alabama Parenting Questionnaire scale, which include setting rules about coming home in evenings, and knowing who an adolescent is friends with (range: 0–40, higher score indicated better supervision) [65]; (4) *Adolescent-Caregiver communication*, as a sum of five items from the Child-Parent Communication Apprehension Scale for use with Young Adults [66]. The scale asks about adolescent-caregiver overall communication as well as communication on sensitive issues, such as medication and sex (range: 0–25); (5) *Education enrolment*, as currently attending school at wave one and two, and currently attending school, university, college, further education or training at wave three; (6) *Days with enough food at home*, as the number of days in the week before the survey with enough food at home.

A full summary of questions and response options relating to sexual risk behaviours and protective factors is provided in Supplementary Table 2, Additional File 1.

#### Covariates

We included eight covariates to account for evidence-based correlates of sexual risk behaviour, the increased probability of some households having access to social grants, and time in our models: HIV status at baseline, age, rural/urban household location, informal/shack housing, number of household residents (including participant), maternal orphanhood, and paternal orphanhood, and an indicator of study wave [55, 67, 68]. For adolescents recruited via municipality health facilities, HIV status was assessed using clinical files. For adolescents recruited in the community, HIV status was measured by self-report during a series of semi-structured questions by trained research assistants at the beginning of each interview, and confirmed in medical records where possible.

### Data analysis

We used four steps in Stata 15. All analyses were disaggregated by gender as there is evidence that protective factors may act differently on HIV risk behaviours amongst girls and boys [35]. First, we described sociodemographic characteristics, protective factors, and HIV risk behaviours overall, by adolescent gender, and by gender, age, and HIV status.

Second, we estimated multivariable associations between our six protective factors and each HIV risk behaviour controlling for covariates. For this we began by using the random effects within-between (REWB) modelling framework described in Bell, Fairbrother, and Jones. 2019 [69]. The value of this framework is its decomposition of time-varying predictors into two distinct constituent sources of variation: (i) between-individual comparison of individuals’ *average* value of a protective factor over study waves; and (ii) within-individual comparisons of individual *deviation* from their average value of a protective factor at each wave. This decomposition enables a comparison of whether between- and within-individual effects differ significantly for predictors of interest. When there is no evidence that they differ, it is valid to model the weighted-average effect of predictors, which is more efficient than separate between- and within-individual effects. Using this framework, for each of our five outcomes, we began by estimating a REWB model (model 1) that included separate within- and between-individual effects for all six time-varying protective factors, and controlled for all eight covariates [69]. Missing values were handled by listwise deletion. We then used the Wald Test for equality to evaluate if pairs of between- and within-individual coefficients differed [70]. Based on the results of these Wald Tests, we then estimated a second REWB model (model 2) that was identical to model 1 but included average effects for time-varying protective factors that showed no evidence of a difference across between- and within-individual coefficients in model 1. To account for risk of type I error from multiple-hypothesis testing in model 2, in addition to p-values, we also estimated sharpened false discovery rate q-values and compared them to the recommended 5% level cut-off [71].

Third, we calculated prevalence ratios (PR), contrasting adjusted probabilities of study outcomes fixing protective factors to ‘0: No’ and ‘1: Yes’ for binary variables and to ‘mean’ and ‘maximum’ for continuous variables. In most cases, maximum scores lay within one standard deviation (SD) of the mean value. Adjusted probabilities and prevalence ratios were calculated overall, and fixing HIV status to either “0. Not living with HIV” and “1. Living with HIV”.

Fourth, as a robustness check for whether variation in protective factors temporally preceded HIV risk behaviours, we evaluated the association between prior (lagged) protective factors and outcomes measured at the subsequent waves. We used the same approach as for our main analysis, except that among boys, we were unable to consider separate within- and between-individual effects for education enrolment because of minimal within-individual variation.

## Results

The sample included 1563 adolescents, and the total number of observations included in the analysis was 4402, Supplementary Fig. 1, Additional file 1. Respondents lost to follow-up in wave two and wave three were older (*p* < 0.001) and lived in larger households (*p* = 0.04) in an urban location (*p* = 0.03), Supplementary Table 3, Additional file 1. Compared to adolescents not living with HIV, those living with HIV at baseline were on average six months younger, more likely to be maternally or paternally orphaned, and lived in smaller households (all *p* < 0.001), Supplementary Table 4, Additional file 1. Median time between first and final interview was 951 days. Missing values for all variables were < 10%, except for sex on substances, which was not measured at wave one, Supplementary Table 5, Additional file 1.

### Summary of descriptive characteristics

Fifty-eight percent of respondents were girls, and 70% were living with HIV, Table 1. The average age of respondents was 15.29 (SD: 3.23), 26% lived in a rural area, 15% lived in informal housing, 41% were maternally orphaned, 34% were paternally orphaned, and the mean household size was 6.39 (SD: 3.00), Table 1. On average, compared to boys, girls were older (*p* < 0.001), lived in larger households (*p* = 0.022), and were more likely to live in informal housing (*p* = 0.003). They were less likely to be enrolled in education (*p* < 0.001), and on average, reported fewer days with enough food at home last week (*p* < 0.001). Between- and within-individual variability in time-varying protective factors was higher among girls, except for within-individual variability in caregiver supervision, which was higher among boys.

**Table 1:** Characteristics of participants overall and by sex over three waves of data collection

	Overall *N* = 1563 Obs = 4402 n %	Girls *N* = 906 Obs = 2530 *n* %	Boys *N* = 657 Obs = 1872 n %	*p*-value^a^
**Sociodemographics**				
Age, mean (SD) [range]	15.29 (3.23) [10-24]	15.78 (3.39) [10-24]	14.63 (2.88) [10-23]	< .001
Living with HIV	3086 (70)	1738 (69)	1348 (72)	0.743
Rural location	1132 (26)	688 (27)	444 (24)	0.088
Informal housing	639 (15)	412 (16)	227 (12)	0.003
Maternal orphan	1796 (41)	983 (39)	813 (43)	0.511
Paternal orphan	1518 (34)	836 (33)	682 (36)	0.053
Household size, mean (SD) [range]	6.39 (3.00) [1-19]	6.51 (3.01) [1-19]	6.22 (2.97) [1-19]	0.022
**Structural factors**				
Number of social grants, mean (SD) [range]	3.16 (2.21) [0–10]	3.23 (2.23) [0–10]	3.07 (2.16) [0–10]	0.833
(Between SD)	(1.88)	(1.92)	(1.83)	
(Within SD)	(1.30)	(1.34)	(1.25)	
Positive caregiving, mean (SD) [range]	18.93 (5.24) [0–24]	18.95 (5.39) [0–24]	18.89 (5.04) [0–24]	0.778
(Between SD)	(3.58)	(3.78)	(3.30)	
(Within SD)	(3.92)	(3.96)	(3.86)	
Caregiver supervision, mean (SD) [range]	33.74 (7.79) [0–40]	33.98 (7.70) [0–40]	33.41 (7.89) [0–40]	0.052
(Between SD)	(5.63)	(5.77)	(5.43)	
(Within SD)	(5.53)	(5.29)	(5.85)	
Adolescent-caregiver communication, mean (SD) [range]	7.24 (2.77) [0–20]	7.21 (2.87) [0–20]	7.28 (2.61) [0–20]	0.220
(Between SD)	(1.81)	(1.91)	(1.68)	
(Within SD)	(2.14)	(2.22)	(2.03)	
Education enrolment	3817 (87)	2040 (81)	1767 (94)	< .001
(Between %)	(93)	(90)	(99)	
(Within %)	(92)	(90)	(96)	
Days with enough food at home last week, mean (SD) [range]	6.53 (1.07) [0–7]	6.46 (1.14) [0–7]	6.62 (0.97) [0–7]	< .001
(Between SD)	(0.76)	(0.82)	(0.67)	
(Within SD)	(0.77)	(0.81)	(0.70)	
**Sexual practices**				
Sexual debut	1516 (34)	1035 (41)	481 (26)	< .001
Multiple sexual partners^b^	625 (14)	346 (14)	279 (15)	0.481
Transactional sex^c^	224 (5)	161 (6)	63 (3)	< .001
Age-disparate sex^d^	238 (5)	182 (7)	56 (3)	< .001
Condomless sex^b^	758 (17)	561 (22)	197 (11)	< .001
Sex on substances^e^	202 (5)	103 (4)	99 (5)	0.156

One thousand five hundred nineteen participants were first interviewed at wave one of data collection, and 44 participants were first interviewed at wave two of data collection

*Abbreviations*: *Obs* Observations, *HIV* Human immunodeficiency virus, *SD* Standard deviation

^a^Estimated from univariable random effects regression to account for clustered nature of data

^b^42 observations missing data

^c^56 observations missing data

^d^120 observations missing data

^e^1563 observations missing data as sex on substances was only measured at waves two and three

**Fig. 1:**
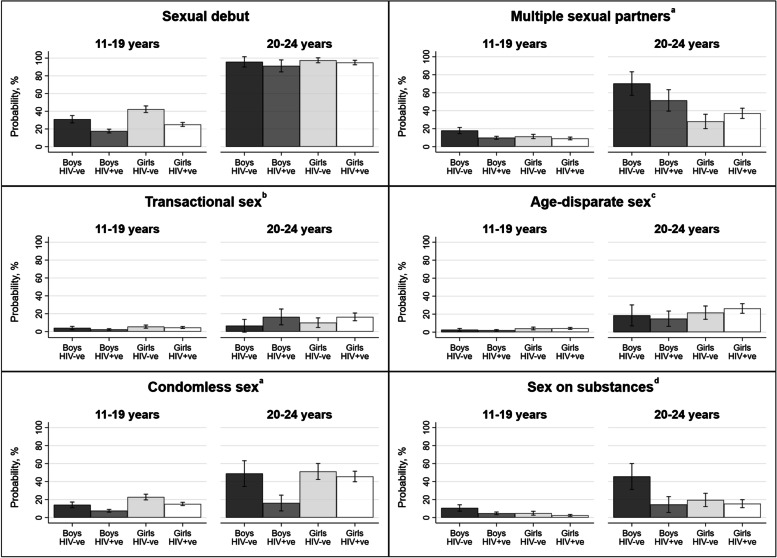
Risk of sexual debut and five HIV risk behaviours by sex, age, and HIV status over three waves of data collection. *N* = 1563, Observations = 4402. ^a^42 observations missing data. ^b^56 observations missing data. ^c^120 observations missing data. ^d^1563 observations missing data upon entry into the study as sex on substances was only measured at waves two and three. Abbreviations: HIV, Human immunodeficiency virus; Adol, Adolescent

Thirty-four percent of the sample had ever had sex. The two most prevalent HIV risk behaviours were multiple sexual partners and condomless sex, followed by transactional sex, age-disparate sex, and sex on substances, Table 1. Girls were more likely to report transactional sex (*p* < 0.001), age-disparate sex (*p* < 0.001), and condomless sex (*p* < 0.001). Compared to at ages 11–19 years, prevalence of all five behaviours was significantly higher at ages 20–25 years (*p* < 0.001), Fig. 1. Compared to their peers not living with HIV, girls living with HIV were significantly less likely to report condomless sex (*p* < 0.001) and sex on substances (*p* = 0.03), and boys living with HIV were less likely to report multiple sexual partners (*p* < 0.001), condomless sex (*p* < 0.001), and sex on substances (*p* < 0.001), Fig. 1. Correlations between study outcomes are summarised in Supplementary Table 6; the prevalence of protective factors by sex, age, and HIV status are summarised in Supplementary Fig. 2; and univariable associations between protective factors and outcomes are summarised in Supplementary Table 7, all in Additional file 1.

### Regression analyses

In models without explanatory variables, values of intra-class correlation (ICC) were > 0.5 for all outcomes − indicating a high correlation of repeated HIV risk behaviours in the same participant across different waves − except for transactional sex in girls (ICC = 0.44) and boys (ICC = 0.20), age-disparate sex in boys (ICC = 0.44), and condomless sex in boys (ICC = 0.33), Supplementary Table 8, Additional file 1.

#### Multivariable associations between hypothesised protective factors and HIV risk behaviours

In model 1, among girls, between- and within-individual coefficients differed significantly for caregiver supervision in relation to multiple sexual partners, condomless sex, and sex on substances; for education enrolment in relation to transactional sex and condomless sex; and for number of days with enough food in relation to transactional sex, Supplementary Table 9, Additional file 1. For each of these relationships, between-individuals, protective factors were associated with lower odds of sexual risk behaviours, whereas within individuals, there was no evidence of association, Supplementary Table 9, Additional file 1. Among boys, between- and within-individual coefficients only differed significantly for education enrolment in relation to condomless sex, Supplementary Table 9, Additional file 1. For each of these relationships, between-individuals, there was no evidence of association, whereas within individuals, education enrolment was associated with higher odds of condomless sex, Supplementary Table 9, Additional file 1.

In model 2, modelling average effects where there was no evidence that between- and within-individual coefficients differed in model 1, among girls, higher positive caregiving scores were associated with lower odds of transactional sex (aOR = 0.94; 95%CI = 0.91, 0.98) and age-disparate sex (aOR = 0.96; 95%CI = 0.93, 1.00); higher caregiver supervision scores were associated with lower odds of transactional sex (aOR = 0.94; 95%CI = 0.92, 0.97), age-disparate sex (aOR = 0.97; 95%CI = 0.94, 0.99); higher adolescent-caregiver communication scores were associated with higher odds of transactional sex (aOR = 1.10; 95%CI = 1.02, 1.18); education enrolment was associated with lower odds of age-disparate sex (aOR = 0.44; 95%CI = 0.26, 0.74); and more days with enough food last week was associated with lower odds of multiple sexual partners (aOR = 0.83; 95%CI = 0.73, 0.95) and transactional sex (aOR = 0.78; 95%CI = 0.68, 0.90), Table 2. Among boys, higher positive caregiving scores were associated with lower odds of transactional sex (aOR = 0.94; 95%CI = 0.90–0.99) and higher odds of condomless sex (aOR = 1.06; 95%CI = 1.02–1.10); higher caregiver supervision scores were associated with lower odds of multiple sexual partners (aOR = 0.93; 95%CI = 0.90, 0.95), transactional sex (aOR = 0.93; 95%CI = 0.90, 0.96), age-disparate sex (aOR = 0.94; 95%CI = 0.91, 0.98), and sex on substances (aOR = 0.92; 95%CI = 0.88, 0.96); education enrolment was associated with higher odds of transactional sex (aOR = 5.56; 95%CI = 1.91, 16.24); and days with enough food last week was associated with lower odds of transactional sex (aOR = 0.77; 95%CI = 0.62, 0.96), Table 2.

**Table 2:** Multivariable associations between hypothesised protective factors and HIV risk practices in girls and boys. Average effects are modelled when there is no evidence that between- and within-individual effects are different. *N* = 1563, Observations = 4402

	Multiple sexual partners			Transactional sex			Age-disparate sex			Condomless sex			Sex on substances^**a**^		
	aOR (95%CI)	naïve *p*-value^b^	sharpened q-value	aOR (95%CI)	naïve *p*-value^b^	sharpened q-value	aOR (95%CI)	naïve *p*-value^b^	sharpened q-value	aOR (95%CI)	naïve *p*-value^b^	sharpened q-value	aOR (95%CI)	naïve *p*-value^b^	sharpened q-value
**Girls**															
Number of social grants															
Between															
Within															
Average	0.99 (0.88–1.12)	0.864	0.976	0.99 (0.86–1.14)	0.851	0.253	1.06 (0.92–1.23)	0.416	0.263	1.08 (0.99–1.19)	0.088	0.258	0.86 (0.70–1.07)	0.181	0.293
Positive caregiving															
Between															
Within															
Average	0.98 (0.95–1.01)	0.283	0.403	0.94 (0.91–0.98)	0.002	0.004	0.96 (0.93–1.00)	0.043	0.061	1.00 (0.98–1.03)	0.921	0.698	1.04 (0.98–1.10)	0.228	0.296
Caregiver supervision															
Between	0.90 (0.86–0.93)	< .001	0.001							0.95 (0.92–0.97)	< .001	0.001	0.86 (0.80–0.92)	< .001	0.001
Within	1.02 (0.99–1.04)	0.123	0.258							1.01 (0.99–1.03)	0.440	0.459	0.96 (0.92–1.00)	0.056	0.202
Average				0.94 (0.92–0.97)	< .001	0.001	0.97 (0.94–0.99)	0.006	0.016						
Adolescent-caregiver communication															
Between															
Within															
Average	0.99 (0.93–1.04)	0.637	0.806	1.10 (1.02–1.18)	0.008	0.007	0.97 (0.91–1.04)	0.357	0.263	0.98 (0.94–1.02)	0.274	0.358	0.93 (0.84–1.03)	0.139	0.293
Education enrolment															
Between										0.42 (0.24–0.72)	0.001	0.005			
Within										0.92 (0.56–1.51)	0.753	0.698			
Average	1.10 (0.70–1.73)	0.669	0.806	1.45 (0.85–2.46)	0.168	0.073	0.44 (0.26–0.74)	0.002	0.013				0.83 (0.39–1.74)	0.618	0.464
Days with enough food															
Between										0.86 (0.71–1.04)	0.117	0.258			
Within										1.08 (0.94–1.25)	0.250	0.358			
Average	0.83 (0.73–0.95)	0.008	0.025	0.78 (0.68–0.90)	< .001	0.003	1.15 (0.95–1.38)	0.145	0.123				0.92 (0.71–1.20)	0.545	0.464
Variance components															
Level 2: In level-1 intercept	2.52			1.15			2.13			1.51			3.43		
Goodness of fit															
AUC	0.96			0.94			0.96			0.93			0.98		
**Boys**															
Number of social grants															
Between															
Within															
Average	1.05 (0.92–1.20)	0.484	1.000	1.02 (0.86–1.21)	0.783	0.354	0.95 (0.77–1.16)	0.613	1.000	1.08 (0.97–1.21)	0.143	0.306	1.05 (0.87–1.27)	0.618	0.981
Positive caregiving															
Between															
Within															
Average	1.00 (0.96–1.05)	0.924	1.000	0.94 (0.90–0.99)	0.030	0.031	1.01 (0.94–1.07)	0.855	1.000	1.06 (1.02–1.10)	0.002	0.015	1.05 (0.98–1.12)	0.155	0.633
Caregiver supervision															
Between															
Within															
Average	0.93 (0.90–0.95)	< .001	0.001	0.93 (0.90–0.96)	< .001	0.001	0.94 (0.91–0.98)	0.002	0.013	0.99 (0.96–1.01)	0.242	0.320	0.92 (0.88–0.96)	< .001	0.001
Adolescent-caregiver communication															
Between															
Within															
Average	1.04 (0.96–1.12)	0.319	1.000	1.03 (0.93–1.12)	0.593	0.311	0.97 (0.87–1.08)	0.622	1.000	0.96 (0.90–1.02)	0.187	0.306	0.95 (0.85–1.06)	0.349	0.871
Education enrolment															
Between										0.69 (0.28–1.75)	0.438	0.487			
Within										2.78 (1.18–6.58)	0.020	0.064			
Average	1.68 (0.80–3.53)	0.168	0.725	5.56 (1.91–16.24)	0.002	0.006	1.52 (0.56–4.12)	0.410	1.000				1.27 (0.50–3.22)	0.619	0.981
Days with enough food															
Between															
Within															
Average	1.03 (0.82–1.29)	0.823	1.000	0.77 (0.62–0.96)	0.019	0.026	1.06 (0.75–1.51)	0.738	1.000	0.99 (0.82–1.18)	0.897	0.513	1.05 (0.76–1.46)	0.766	1.000
Variance components															
Level 2: In level-1 intercept	2.69			0.00			1.70			0.66			3.18		
Goodness of fit															
AUC	0.97			0.86			0.96			0.88			0.98		

Coefficients for additional covariates are available in Additional file 1: Table 10

*Abbreviations*: *aOR* Adjusted odds ratio, *CI* Confidence interval, *AUC* Area under curve

^a^Sex on substances was only measured at wave two and wave three

^b^Wald *p*-value for significance. A significant p-value indicates that a coefficient is significantly different from 1.00. The AUC statistic ranges from 0 to 1 and gives an idea of how well a model is able to distinguish between positive and negative outcomes. The higher the AUC, the better the model is at correctly classifying outcomes

In model 2, modelling between- and within-individual effects concurrently where there was evidence that these terms differed in model 1, among girls, between-individuals, higher caregiver supervision scores were associated with lower odds of multiple sexual partners (aOR = 0.90; 95%CI = 0.86, 0.93), condomless sex (aOR = 0.95; 95%CI = 0.92, 0.97), and sex on substances (aOR = 0.86; 95%CI = 0.80, 0.92); and education enrolment was associated with lower odds of condomless sex (aOR = 0.42; 95%CI = 0.24, 0.72), Table 2. Within-individuals, there was no evidence of association between these protective factors and outcomes, Table 2. Among boys, there was no evidence of association between protective factors and sexual risk behaviours either between-individuals, Table 2. Within-individuals, education enrolment was associated with higher odds of condomless sex (aOR = 2.78; 95%CI = 1.18, 6.58), Table 2.

Controlling for multiple hypothesis testing using sharpened q-values, we could not rule out that among girls, the average association between positive caregiving and age-disparate sex was a false discovery, Table 2. Among boys, we could not rule out that the within-individual association between education enrolment and higher odds of condomless sex was a false discovery, Table 2.

Model 2 adjusted odds ratios for the covariates HIV status, rural location, informal housing, household size, maternal orphanhood, paternal orphanhood, age, and study wave are summarised in Supplementary Table 10, Additional file 1.

#### Prevalence ratios contrasting adjusted probabilities of HIV risk behaviours at selected values of protective factors

**Fig. 2:**
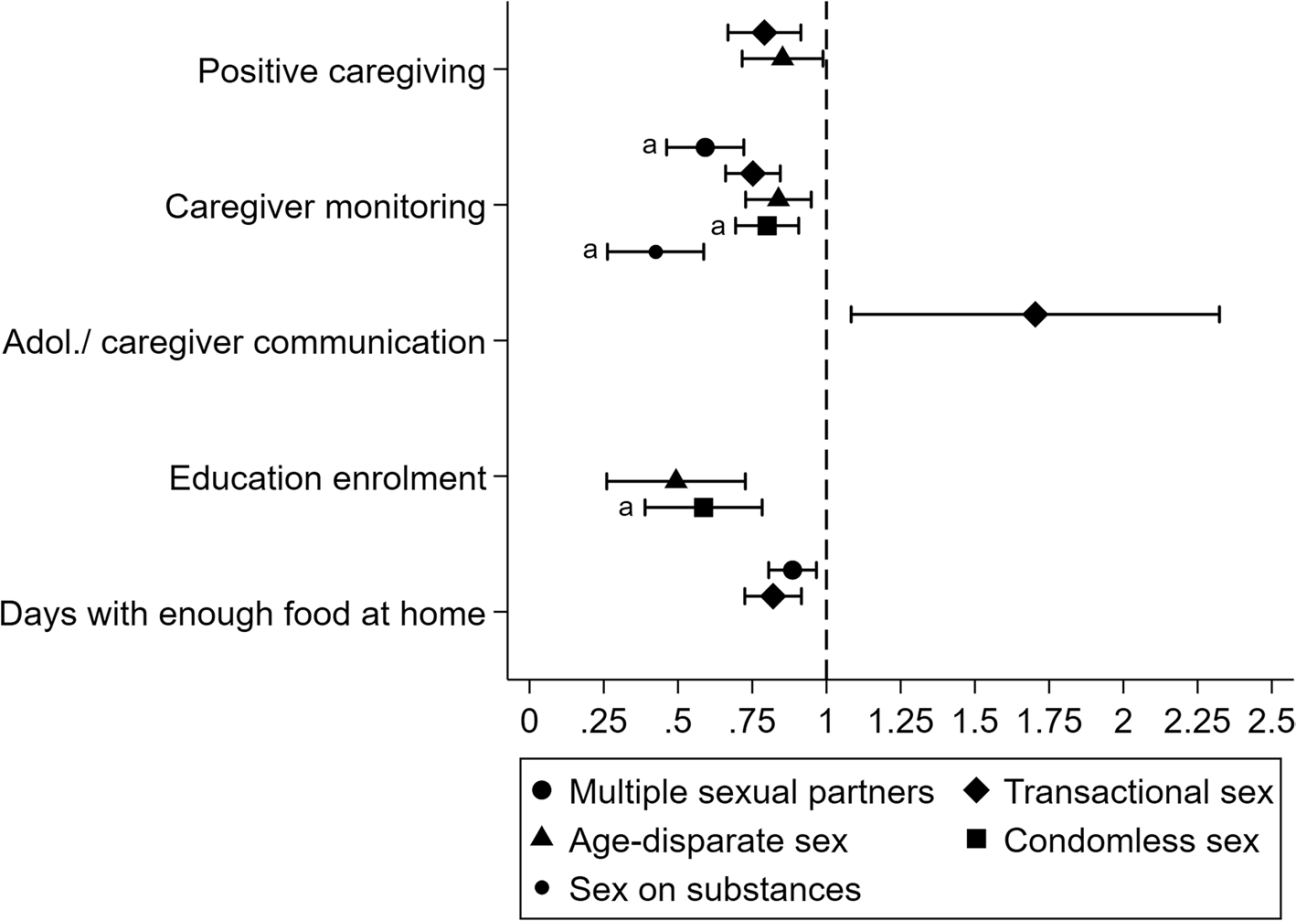
Prevalence ratios contrasting the adjusted probability of HIV risk behaviours for two scenarios among girls: (i) at the mean of continuous protective factors, or in the absence of education enrolment; and (ii) at the maximum of continuous protective factors, or in the presence of education enrolment. We only calculated prevalence ratios where there was evidence of significant associations between protective factors and HIV risk behaviours. Values used to build Fig. 2 are summarised in Additional file 1: Supplementary Table 13. ^a^Predictions are based on between-individual effects rather than average effects

**Fig. 3:**
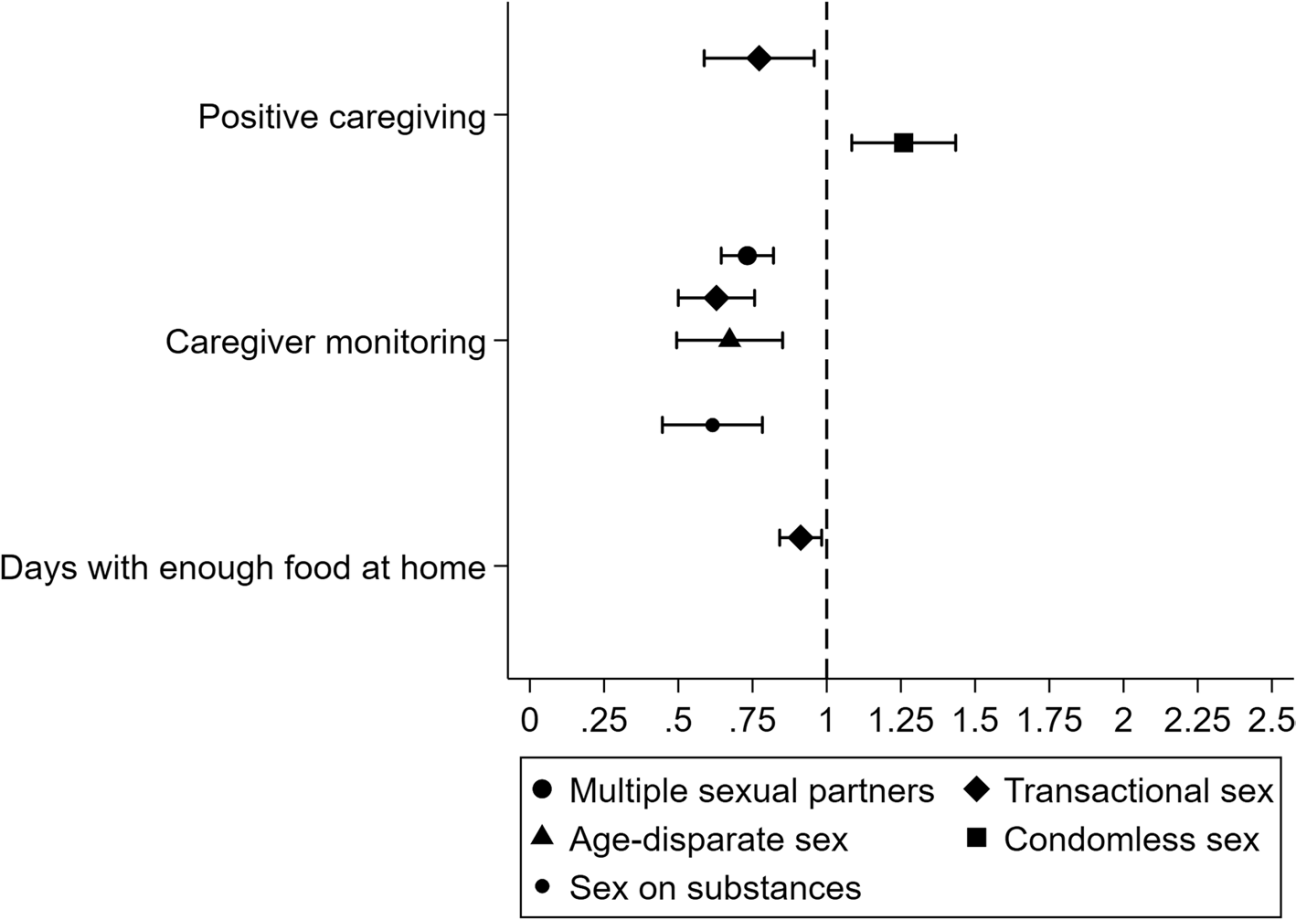
Prevalence ratios contrasting the adjusted probability of HIV risk behaviours for two scenarios among boys: (i) at the mean of continuous protective factors; and (ii) at the maximum of continuous protective factors. We only calculated prevalence ratios where there was evidence of significant associations between protective factors and odds of HIV risk behaviours. There was no evidence that estimated prevalence ratios contrasting the adjusted probability of transactional sex or condomless sex in the absence and presence of education enrolment were significant, so we did not plot them. Values used to build Fig. 3 are summarised in Supplementary Table 13, Additional file 1

We summarise prevalence ratios comparing adjusted probabilities for each of our HIV risk behaviours at selected values of significant protective factors among girls in Fig. 2 and boys in Fig. 3.

#### Multivariable associations between lagged hypothesised protective factors and HIV risk behaviours

Lagged model 1 between- and within-individual coefficients are reported in Supplementary Table 11, Additional file 1. In lagged model 2, modelling average effects where there was no evidence that between- and within-individual coefficients differed, among girls, higher prior caregiver supervision scores were associated with lower odds of subsequent multiple sexual partners (aOR = 0.95; 95%CI = 0.93, 0.98), and condomless sex (aOR = 0.96; 95%CI = 0.94, 0.98); higher prior number of days with enough food was associated with lower odds of subsequent multiple sexual partners (aOR = 0.81; 95%CI = 0.69, 0.96) and condomless sex (aOR = 0.82; 95%CI = 0.72, 0.93), Supplementary Table 12, Additional file 1. Among boys, higher prior adolescent-caregiver communication scores were associated with higher odds of sex on substances (aOR = 1.13; 95%CI = 1.00, 1.28), Supplementary Table 12, Additional file 1.

In lagged model 2, modelling between- and within-individual effects concurrently where there was evidence of a significant difference between these terms, among girls, between-individuals, higher prior caregiver supervision scores were associated with lower odds of subsequent transactional sex (aOR = 0.94; 95%CI = 0.89, 0.99), age-disparate sex (aOR = 0.95; 95%CI = 0.91, 0.99), and sex on substances (aOR = 0.90; 95%CI = 0.85, 0.96), Supplementary Table 12, Additional file 1. Within-individuals, there was no evidence of an association between protective factors and sexual risk behaviours, Supplementary Table 12, Additional file 1. Among boys, between-individuals, higher prior caregiver supervision scores were associated with lower odds of subsequent multiple sexual partners (aOR = 0.94; 95%CI = 0.89, 0.99), transactional sex (aOR = 0.94; 95%CI = 0.90, 0.99), and sex on substances (aOR = 0.92; 95%CI = 0.86, 0.98), Supplementary Table 12, Additional file 1. Within-individuals, higher prior caregiver supervision scores were associated with lower odds of subsequent transactional sex (aOR = 1.07; 95%CI = 1.02, 1.13), Supplementary Table 12, Additional file 1.

Controlling for multiple hypothesis testing using sharpened q-values, we could not rule out that among girls, the lagged between-individual associations between caregiver supervision and lower odds of transactional sex and age-disparate sex were false discoveries, Supplementary Table 12, Additional file 1. Among boys, we could not rule out that all the observed associations between prior protective factors and subsequent sexual risk behaviours were false discoveries.

## Discussion

This study found a high prevalence of five HIV risk behaviours in a large cohort of adolescents in South Africa’s Eastern Cape. Rates of condomless sex were much higher than the UNAIDS target of no more than 5% for priority groups [72, 73], and young men reported high rates of multiple sexual partners [74]. Compared to peers not living with HIV, girls living with HIV were significantly less likely to engage in condomless sex, and boys living with HIV were less likely to engage in both condomless sex and sex on substances. In gender-stratified analyses, we found that among girls, increased positive caregiving, caregiver supervision, and days with enough food at home, were each associated with lower probability of multiple HIV risk behaviours, and education enrolment was associated with lower probability of age-disparate sex. Additionally, prior increases in caregiver supervision and number of days with enough food were associated with lower odds of subsequently experiencing multiple sexual partners and condomless sex, suggesting that the protective influence of these factors may be sustained, or develop over time for these outcomes. Among boys, increased caregiver supervision was associated with lower probability of multiple HIV risk behaviours, and increased positive caregiving and days with enough food were associated with lower probability of transactional sex. There was no evidence that the influence of protective factors is sustainted, or develops over time in this group.

Our analysis applies rigorous statistical methods to three waves of data to investigate relationships between six protective factors and five HIV risk behaviours simultaneously, further unpacking differential associations for girls and boys. In girls, associations between education enrolment and age-disparate sex are consistent with previous analyses and theories supporting a positive relationship between schooling and safer sexual networks and more negotiating power among adolescent girls [25, 75–79]. The observed association between days with enough food at home and transactional sex is supported by the ‘sex for basic needs’ paradigm of transactional sex [80], and the concurrent relationship between this factor and both multiple sexual partnership and transactional sex matches qualitative reports of the influence of poverty on sexual behaviours among adolescent girls [27]. Associations between caregiver supervision and transactional sex and age-disparate sex are in line with theories suggesting that setting rules and monitoring peer-relationships can act as a ‘protective shield’, promoting the internalization of norms that foster healthy behaviours, mitigating sensation-seeking and impulsive decision-making, and deterring affiliation with deviant peers [81–84]. Among boys, the relationship between caregiver supervision and four out of five study outcomes may be linked to masculine norms of independence and sexuality driving HIV risk in this population, and caregivers’ gendered perceptions that independence should be encouraged among adolescent boys, while adolescent girls should remain restricted and protected [85, 86].

Our findings support continued emphasis on structural interventions for enhancing the effectiveness of core HIV prevention programmes [87]. Among girls, the range of protective factors associated with HIV risk behaviours validates the need for comprehensive multi-component prevention approaches in contexts with sufficient resources [42, 68]. Comparing across protective factors, the strong association between education enrolment and age-disparate sex suggests that, in settings with limited resources, interventions should be designed around a core focus to support adolescent girls’ education [88]. Existing evidence suggests that cash transfers are an effective way of achieving this [89]. Where possible, efforts should also aim to improve support for girls’ education by addressing unaffordable fees [90], poor-quality education and associated learning backlogs [91, 92], early motherhood [93], and low morale for future employment [94]. The finding that caregiver supervision and number of days with enough food may only be associated with small reductions in HIV risk behaviours indicates that there should be careful consideration of cost-effective approaches for layering multicomponent interventions. Among boys, the finding that caregiver supervision may be such a major protective factors points to a more singular approach for reducing HIV risk. This could focus on encouraging more sustained supervision either by caregivers or other community members such as ‘social fathers’ [39, 95]. Such interventions should be sensitive to local norms around masculinity and transitions to manhood [96]. Finally, findings that HIV risk behaviours remain high in both adolescents living with HIV and their peers not living with HIV supports the continued need for population-wide interventions aimed at preventing both new infections and onwards transmission.

This study’s use of three waves of data spanning an average of two and a half years for each participant enabled us to use advanced statistical models to unpack between- and within-individual associations linking protective factors and HIV risk behaviours. Nevertheless, there is still a risk of confounding from unmeasured time-varying factors for both between- and within-individual associations, and estimated associations should not be interpreted causally. The study’s thorough sampling strategy and small loss to follow-up minimises risk of selection bias among participants living with HIV. Although participants not living with HIV were recruited through invitation, the large number of respondents should also reduce selection bias in this group. Self-reported items may be subject to social desirability and recall bias, particularly those relating to sensitive topics, and participant subjectivity may also be a source of measurement error [97]. Our use of questions with categorical responses (e.g. Yes/ No) rather than continuous (e.g. number of times) may have mitigated recall bias in sexual risk behaviours [98]. Since almost all participants in our study reported receipt of at least one social grant (~ 91%) we were unable to robustly evaluate the association between this protective factor and HIV risk behaviours. Similarly, among boys, since almost all reported being enrolled in education (94%) with little between- or within-individual variation, we were limited in our ability to robustly evaluate the association between this protective factor and rarer sexual risk behaviours. Further research should aim to validate our results in the future.

Building on this study, future quantitative mediation analysis and qualitative interviews could valuably inform the mechanisms via which protective factors act on HIV risk behaviours, and provide stronger causal claim for associations identified in this study. Plausible pathways could include stronger affiliation with a positive peer group, safer sexual networks, and greater negotiating power [99, 100]. Our finding that even when enrolled in education, adolescent girls may still experience a high probability of condomless sex, highlights an urgent needed to identify other protective factors able to address this key sexual practice [101]. Further, evidence indicates that addressing community disorder, caregiver stress, and young men's social inclusion could be valuable candidates for reducing HIV transmission in the future [102].

## Conclusions

Adolescent girls enrolled in education with greater food security, and adolescent girls and boys experiencing positive and supervisory caregiving are less likely to engage in sexual risk behaviours and experiences linked to HIV transmission. Investments in effective structural interventions to enhance these factors among young people are likely to translate into crucial progress in reducing HIV incidence.

## Supplementary Information

**Additional file 1: Supplementary Table 1.** Study STROBE statement. **Supplementary Table 2.** Summary of questionnaire items and response options for protective factors and HIV risk practices. **Supplementary Table 3.** Baseline characteristics of respondents by loss to follow-up. **Supplementary Table 4.** Baseline characteristics of study respondents by HIV status. **Supplementary Table 5.** Summary of missing values. **Supplementary Figure 1.** Flow chart of respondents in Mzantsi Wakho cohort. **Supplementary Figure 2.** Prevalence of six protective factors by sex, age, and HIV status. *N*=1563, Observations=4402. **Supplementary Table 6.** Intercorrelations between study outcomes. *N*=1563, Observations=4402. **Supplementary Table 7.** Univariable associations between hypothesised protective factors and HIV risk practices in girls and boys. *N*=1563, Observations=4402. **Supplementary Table 8.** Intra-class correlations for HIV risk practices from null models (i.e. with no predictors). **Supplementary Table 9.** Multivariable associations between hypothesised protective factors and HIV risk practices in girls and boys. Between- and within-individual effects are modelled separately for all protective factors. *N*=1563, Observations=2883. **Supplementary Table 10.** Summary of adjusted odds ratios for additional covariates included in models investigating multivariable associations between hypothesised protective factors and HIV risk practices in girls and boys. **Supplementary Table 11.** Multivariable lagged associations between hypothesised protective factors and HIV risk practices in girls and boys. Between- and within-individual effects are modelled separately for all protective factors. *N*=1563, Observations=2883. **Supplementary Table 12.** Multivariable lagged associations between hypothesised protective factors and HIV risk practices in girls and boys. Average effects are modelled when there is no evidence that within- and between-individual effects are different. *N*=1563, Observations=2883. **Supplementary Table 13.** Adjusted probabilities and probability ratios for HIV risk practices at the mean and maximum of selected protective factors. Adjusted probabilities were estimated with all covariates at observed values.

## Data Availability

The data that support the findings of this study are available from the corresponding author upon reasonable request.

## Abbreviations

AIDS: Acquired immunodeficiency syndrome
ART: Antiretroviral therapy
CI: Confidence interval
DREAMS: Determined, Resilient, Empowered, AIDS-free, Mentored, and Safe
HIV: Human immunodeficiency virus
ICC: Intra-class correlation
OR: Odds ratio
PR: Prevalence ratio
PEPFAR: United States President’s Emergency Plan for AIDS Relief
PREPARE: Promoting sexual and reproductive health among adolescents in Southern and Eastern Africa
REWB: Random effects within-between
SASSA: South African Social Security Agency
SD: Standard deviation
UNAIDS: The United Nations Programme on HIV/AIDS
UNICEF: The United Nations Children’s Fund
USAID: The United States Agency for International Development
