# Supplementary material for: Protective factors for adolescent sexual risk behaviours and experiences linked to HIV infection in South Africa: a three-wave longitudinal analysis of caregiving, education, food security, and social protection
Source: BMC Public Health. 2023 Jul 29;23:1452. doi: 10.1186/s12889-023-16373-5 (PMC10386676; doi:10.1186/s12889-023-16373-5)
Supplement: Supplementary file 1 — Additional file 1: Supplementary Table 1. Study STROBE statement. Supplementary Table 2. Summary of questionnaire items and response options for protective factors and HIV risk practices. Supplementary Table 3. Baseline characteristics of respondents by loss to follow-up. Supplementary Table 4. Baseline characteristics of study respondents by HIV status. Supplementary Table 5. Summary of missing values. Supplementary Figure 1. Flow chart of respondents in Mzantsi Wakho cohort. Supplementary Figure 2. Prevalence of six protective factors by sex, age, and HIV status. N=1563, Observations=4402. Supplementary Table 6. Intercorrelations between study outcomes. N=1563, Observations=4402. Supplementary Table 7. Univariable associations between hypothesised protective factors and HIV risk practices in girls and boys. N=1563, Observations=4402. Supplementary Table 8. Intra-class correlations for HIV risk practices from null models (i.e. with no predictors). Supplementary Table 9. Multivariable associations between hypothesised protective factors and HIV risk practices in girls and boys. Between- and within-individual effects are modelled separately for all protective factors. N=1563, Observations=2883. Supplementary Table 10. Summary of adjusted odds ratios for additional covariates included in models investigating multivariable associations between hypothesised protective factors and HIV risk practices in girls and boys. Supplementary Table 11. Multivariable lagged associations between hypothesised protective factors and HIV risk practices in girls and boys. Between- and within-individual effects are modelled separately for all protective factors. N=1563, Observations=2883. Supplementary Table 12. Multivariable lagged associations between hypothesised protective factors and HIV risk practices in girls and boys. Average effects are modelled when there is no evidence that within- and between-individual effects are different. N=1563, Observations=2883. Supplementary Table 13. [file 12889_2023_16373_MOESM1_ESM.docx]

**Additional file 1**

**Supplementary Table 1. Study STROBE statement.**

|  | Item No. | Recommendation | Page No. |
| --- | --- | --- | --- |
| Title and abstract | 1 | (a) Indicate the study’s design with a commonly used term in the title or the abstract | 1 |
|  |  | (b) Provide in the abstract an informative and balanced summary of what was done and what was found | 2-3 |
| Introduction |  |  |  |
| Background/rationale | 2 | Explain the scientific background and rationale for the investigation being reported | 3-5 |
| Objectives | 3 | State specific objectives, including any prespecified hypotheses | 5 |
| Methods |  |  |  |
| Study design | 4 | Present key elements of study design early in the paper | 6-7 |
| Setting | 5 | Describe the setting, locations, and relevant dates, including periods of recruitment, exposure, follow-up, and data collection | 6-7 |
| Participants | 6 | Give the eligibility criteria, and the sources and methods of selection of participants. Describe methods of follow-up | 6 |
| Variables | 7 | Clearly define all outcomes, exposures, predictors, potential confounders, and effect modifiers. Give diagnostic criteria, if applicable | 7-9 |
| Data sources/ measurement | 8 | For each variable of interest, give sources of data and details of methods of assessment (measurement). Describe comparability of assessment methods if there is more than one group | 7-9 |
| Bias | 9 | Describe any efforts to address potential sources of bias | 10 |
| Study size | 10 | Explain how the study size was arrived at | 6 |
| Quantitative variables | 11 | Explain how quantitative variables were handled in the analyses. If applicable, describe which groupings were chosen and why | 9-10 |
| Statistical methods | 12 | (a) Describe all statistical methods, including those used to control for confounding | 9-10 |
|  |  | (b) Describe any methods used to examine subgroups and interactions | 9-10 |
|  |  | (c) Explain how missing data were addressed | 9 |
|  |  | (d) If applicable, explain how loss to follow-up was addressed | 9 |
|  |  | (e) Describe any sensitivity analyses | NA |
| Results |  |  |  |
| Participants | 13 | (a) Report numbers of individuals at each stage of study—eg numbers potentially eligible, examined for eligibility, confirmed eligible, included in the study, completing follow-up, and analysed | 10 |
|  |  | (b) Give reasons for non-participation at each stage | 10 |
|  |  | (c) Consider use of a flow diagram | Supplementary Figure 2 |
| Descriptive data | 14 | (a) Give characteristics of study participants (eg demographic, clinical, social) and information on exposures and potential confounders | 11 |
|  |  | (b) Indicate number of participants with missing data for each variable of interest | Supplementary Table 5 |
|  |  | (c) Summarise follow-up time (eg, average and total amount) | 10 |
| Outcome data | 15 | Report numbers of outcome events or summary measures over time | 11 |
| Main results | 16 | (a) Give unadjusted estimates and, if applicable, confounder-adjusted estimates and their precision (eg, 95% confidence interval). Make clear which confounders were adjusted for and why they were included | Supplementary Table 7 |
|  |  | (b) Report category boundaries when continuous variables were categorized | 14 |
|  |  | (c) If relevant, consider translating estimates of relative risk into absolute risk for a meaningful time period | 14 |
| Other analyses | 17 | Report other analyses done—eg analyses of subgroups and interactions, and sensitivity analyses | 14-15 |
| Discussion |  |  |  |
| Key results | 18 | Summarise key results with reference to study objectives | 15 |
| Limitations | 19 | Discuss limitations of the study, taking into account sources of potential bias or imprecision. Discuss both direction and magnitude of any potential bias | 17 |
| Interpretation | 20 | Give a cautious overall interpretation of results considering objectives, limitations, multiplicity of analyses, results from similar studies, and other relevant evidence | 18 |
| Generalisability | 21 | Discuss the generalisability (external validity) of the study results |  |

**Supplementary Table 2. Summary of questionnaire items and response options for protective factors and HIV risk practices.**

|  | **Questionnaire items and response options** |
| --- | --- |
| **Protective factors** |  |
| Number of social grants | Number of social grants received by the adolescent’s household including: child support grant, foster care grant, disability grant, pension grant, and care dependency grant.  "Are you or your household receiving any grants?"  "How many foster care grants does your household receive?"  **Response options: Yes; No**  "How many child support grants does your household receive?"  "How many foster care grants does your household receive?"  "How many disability grants does your household receive?"  "How many pension grants does your household receive?"  "How many care dependency grants does your household receive?" |
| Education enrolment | Currently attending school or university  "What kind of school do you go to?"  **Response option: Primary or secondary school; University, college, FET or another tertiary institution; I am not in school** |
| Days without enough food at home last week | "Sometimes kids don't have enough food in their home. How many days in the seven days did you not have enough food in your home?" |
| Positive caregiving | Alabama Parenting Questionnaire scale, positive parenting subscale, sum of 6 items. Scores range from 0 to 24, with higher scores reflect better/more positive parenting by parents/caregivers.  "Please answer these questions about stuff that happened at home in the past two months"  1. Your parents or caregiver says you have done something well.  2. Your parent or caregiver compliments you when you have done something well.  3. Your parent or caregiver praises you for behaving well.  4. Your parent or caregiver tells you that they like it when you help out around the house.  5. Your parent or caregiver rewards or gives something extra to you for behaving well.  6. Your parents or caregivers hug you or kiss you when you have done something well.  **Response options: Never; Rarely; Sometimes; Often; Always** |
| Caregiver supervision | Alabama Parenting Questionnaire scale, poor monitoring & supervision subscale, sum of 10 items. Scores range from 0 to 40. Higher scores usually reflect worse monitoring and supervision by parents/caregivers, but we reversed the scale in this study such that higher scores reflect better/ more monitoring and supervision.  "Please answer these questions about stuff that happened at home in the past two months"  1. You go out without a set time to be home.  2. You stay out in the evening past the time you are supposed to be at home.  3. You fail to leave a note or let your parent or caregiver know where you are going.  4. Your parent or caregiver does not know who you are friends with.  5. You go out after dark without an adult with you.  6. Your parent or caregiver gets so busy that they forget where you are and what you are doing.  7. You stay out later than you are supposed to and your caregiver doesn’t know it.  8. Your parent or caregiver leaves the house and doesn’t tell you where they are going.  9. You come home from school more than an hour past the time your parent or caregiver expects you to be home.  10. You are at home without an adult with you.  **Response options: Never; Rarely; Sometimes; Often; Always** |
| Adolescent-caregiver communication | Child-Parent Communication Apprehension Scale for use with Young Adults, sum of five items. Higher scores reflect better communication.  "We’d like to know more about how you feel talking to your parents or caregivers. Could you tell us about how much you’ve shared with your caregiver in the past two months?"  1. I have no fear in discussing problems with my parent or caregiver.  2. I am comfortable talking about sex or medication with my parents or caregivers.  3. I am relaxed with my parent or caregiver, I can talk to them openly.  4. When I talk to my parent or caregiver, I am anxious and careful about what I say. (reverse-coded)  5. I have no fear telling my parents or caregivers exactly how I feel.  **Response options: Strongly disagree; Disagree; Don’t know; Agree; Strongly agree** |
| **HIV risk practices** |  |
| Multiple sexual partners | "How many people have you had sex with in the past year?" |
| Transactional sex | "Have you had any of these presents given to you because you had sex with someone, or did you decide to have sex with someone in exchange for these gifts?"  1. Receipt of money  2. Drinks  3. Clothes  4. Cell phone airtime  5. A place to stay  6. Lifts in a car/taxi  7. Better marks at school  8. School fees  9. Food  10. Or anything else for having sex with someone.  **This question was drawn from the National Survey of Risk Behaviour Amongst Young South Africans.** |
| Age-disparate sex | Think about the oldest person you had sex with in the last year. Was he or she more than 5 years older than you?" |
| Condomless sex | "In the last year, how often did you use condoms for the whole time that you were having sex?"  **Response options: Never, Less than half the time, Half the time, More than half the time, Every time** |
| Sex on substances | In the last year, how many times have you had sex when you were drunk or smoking dagga or any other drugs? |

Abbreviations: HIV, human immunodeficiency virus.

**Supplementary Table 3. Baseline characteristics of respondents by loss to follow-up.**

|  | **Lost to follow-up data collection** | |  |
| --- | --- | --- | --- |
|  | **No**  **(n=1392)** | **Yes**  **(n=171)** | **p-value** |
| **Sociodemographic characteristics** |  |  |  |
| Age, mean (SD) | 13.97 (3.12) | 14.89 (3.12) | <.001 |
| Girls | 803 (58) | 103 (60) | 0.52 |
| HIV status | 963 (69) | 117 (68) | 0.84 |
| Rural location | 385 (28) | 34 (20) | 0.03 |
| Informal housing | 243 (17) | 30 (18) | 0.98 |
| Household size, mean (SD) | 7.12 (4.17) | 7.91 (8.27) | 0.04 |
| Maternal orphan | 514 (37) | 63 (37) | 0.98 |
| Paternal orphan | 384 (28) | 53 (31) | 0.35 |
| **Protective factors** |  |  |  |
| Number of social grants, mean (SD) [range] | 3.29 (2.15) [0-10] | 2.94 (3.01) [0-10] | 0.05 |
| Education enrolment | 1,281 (92) | 153 (89) | 0.22 |
| Days with enough food at home last week, mean (SD) [range] | 6.49 (1.24) [0-7] | 6.56 (1.15) [0-7] | 0.54 |
| Positive caregiving, mean (SD) [range] | 19.73 (4.92) [0-24] | 19.13 (5.23) [0-24] | 0.13 |
| Caregiver supervision, mean (SD) [range] | 33.60 (8.62) [0-40] | 32.52 (8.64) [0-40] | 0.12 |
| Adolescent-caregiver communication, mean (SD) [range] | 7.21 (2.59) [0-15] | 7.06 (3.07) [0-15] | 0.49 |

Abbreviations: HIV, human immunodeficiency virus; SD, standard deviation.

**Supplementary Table 4. Baseline characteristics of study respondents by HIV status.**

|  | **Not living with HIV**  **(n=483)** | **Living with HIV**  **(n=1,080)** | **p-value** |
| --- | --- | --- | --- |
| **Sociodemographic characteristics** |  |  |  |
| Age, mean (SD) | 14.49 (3.10) | 13.88 (3.13) | <.001 |
| Girls | 297 (61) | 609 (56) | 0.06 |
| Rural location | 139 (29) | 280 (26) | 0.24 |
| Informal housing | 75 (16) | 198 (18) | 0.19 |
| Household size, mean (SD) | 8.15 (7.04) | 6.78 (3.24) | <.001 |
| Maternal orphan | 103 (21) | 474 (44) | <.001 |
| Paternal orphan | 110 (23) | 327 (30) | <.001 |
| **Protective factors** |  |  |  |
| Number of social grants, mean (SD) [range] | 3.46 (2.35) [0-10] | 3.16 (2.21) [0-10] | 0.02 |
| Positive caregiving, mean (SD) [range] | 19.59 (5.04) [0-24] | 19.70 (4.92) [0-24] | 0.69 |
| Caregiver supervision, mean (SD) [range] | 33.01 (7.86) [0-40] | 33.69 (8.94) [0-40] | 0.15 |
| Adolescent-caregiver communication, mean (SD) [range] | 7.02 (2.84) [0-20] | 7.27 (2.54) [0-20] | 0.08 |
| Education enrolment | 448 (93) | 986 (91) | 0.32 |
| Days with enough food at home last week, mean (SD) [range] | 6.57 (1.06) [0-7] | 6.47 (1.30) [0-7] | 0.15 |

Abbreviations: HIV, human immunodeficiency virus; SD, standard deviation.

**Supplementary Table 5. Summary of missing values.**

|  | **Missing values**  **N=4689** |
| --- | --- |
| Sex | 287 (6) |
| Age | 287 (6) |
| HIV status | 287 (6) |
| HIV awareness | 291 (6) |
| Rural | 319 (7) |
| Informal house | 290 (6) |
| Household size | 287 (6) |
| Maternal orphan | 287 (6) |
| Paternal orphan | 287 (6) |
| Multiple sexual partners | 329 (6) |
| Transactional sex | 343 (7) |
| Age-disparate sex | 407 (9) |
| Condomless sex | 329 (7) |
| Sex on substances^a^ | 1848 (39) |
| Number of social grants | 297 (6) |
| Education enrolment | 387 (8) |
| Days with enough food last week | 287 (6) |
| Positive caregiving | 287 (6) |
| Caregiver supervision | 287 (6) |
| Adolescent-caregiver communication | 287 (6) |

^a^Sex on substances was only measured at wave two and wave three.

Abbreviations: HIV, human immunodeficiency virus.

**
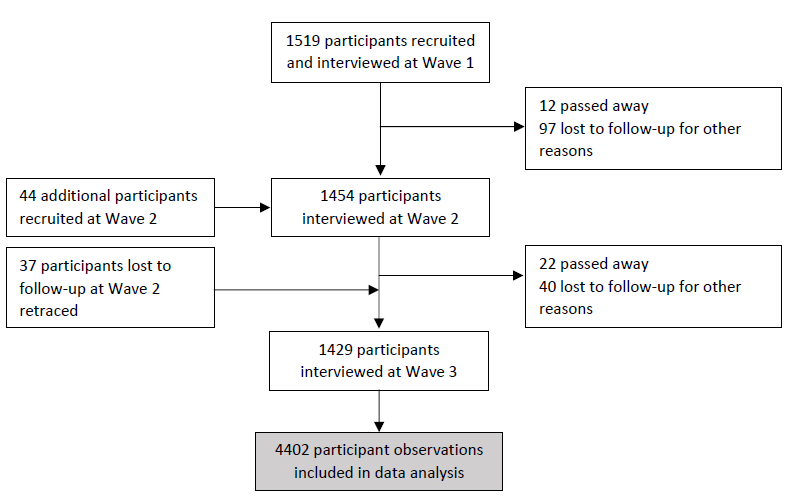
**

**Supplementary Figure 1. Flow chart of respondents in Mzantsi Wakho cohort.**


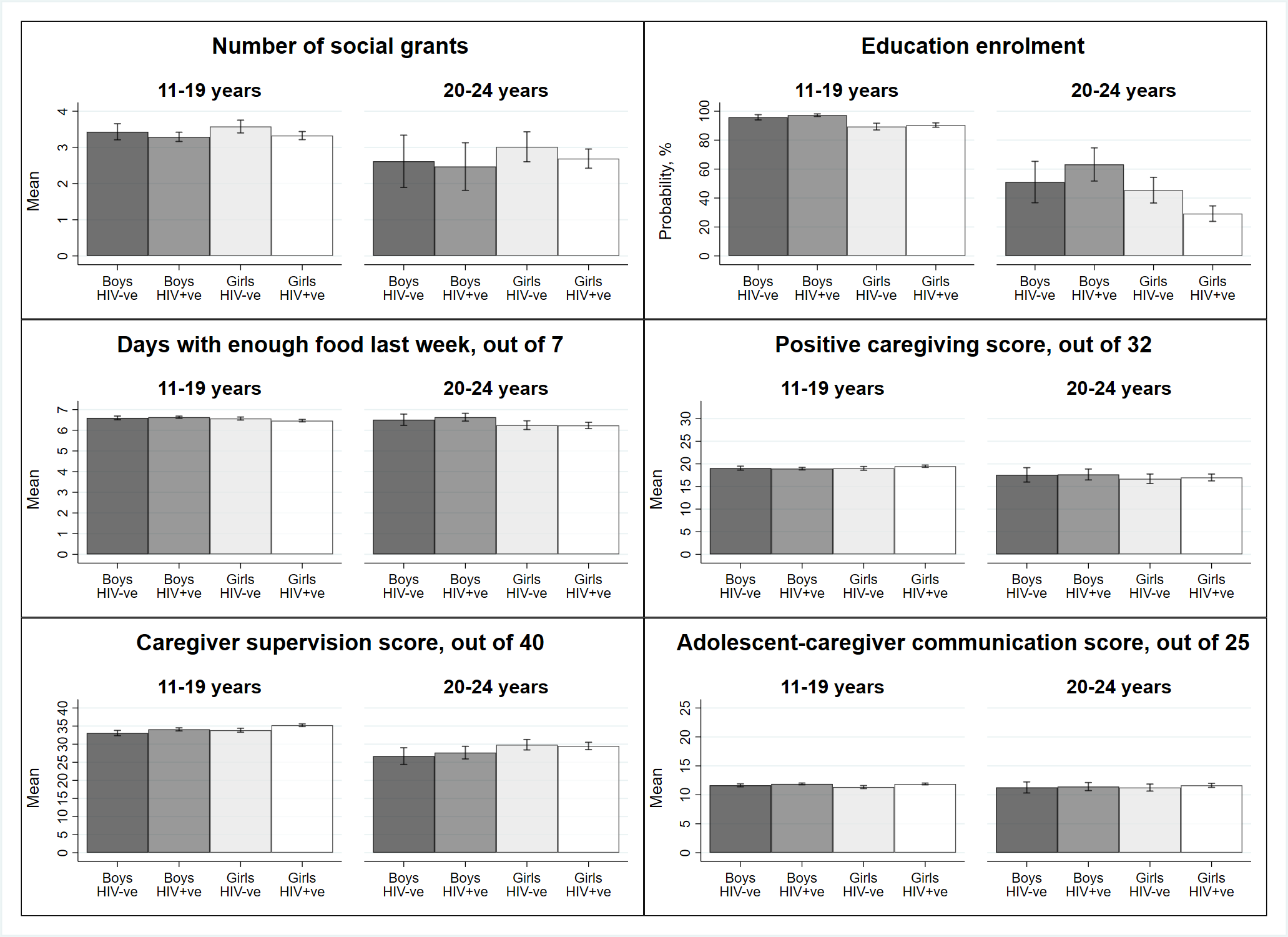


**Supplementary Figure 2. Prevalence of six protective factors by sex, age, and HIV status. N=1563, Observations=4402.**

Abbreviations: HIV, human immunodeficiency virus.

**Supplementary Table 6. Intercorrelations between study outcomes. N=1563, Observations=4402.**

|  | **n (%)** | **Correlations** | | | | |
| --- | --- | --- | --- | --- | --- | --- |
|  |  | **1** | **2** | **3** | **4** | **5** |
| **Girls (Obs=2530)** |  |  |  |  |  |  |
| 1. Multiple sexual partners | 346 (14) | 1.000 |  |  |  |  |
| 2. Transactional sex | 161 (6) | 0.286 | 1.000 |  |  |  |
| 3. Age-disparate sex | 182 (7) | 0.246 | 0.113 | 1.000 |  |  |
| 4. Condomless sex | 561 (22) | 0.329 | 0.192 | 0.256 | 1.000 |  |
| 5. Sex on substances^a^ | 103 (4) | 0.263 | 0.153 | 0.111 | 0.214 | 1.000 |
| **Boy (Obs=1872)** |  |  |  |  |  |  |
| 1. Multiple sexual partners | 279 (15) | 1.000 |  |  |  |  |
| 2. Transactional sex | 63 (3) | 0.329 | 1.000 |  |  |  |
| 3. Age-disparate sex | 56 (3) | 0.324 | 0.285 | 1.000 |  |  |
| 4. Condomless sex | 197 (11) | 0.281 | 0.138 | 0.171 | 1.000 |  |
| 5. Sex on substances^a^ | 99 (5) | 0.503 | 0.235 | 0.267 | 0.259 | 1.000 |

Coefficients are pairwise Spearman correlations. Moderate correlation: ±0.30 = minimal, ±0.40 = important, and ±.50 = practically significant. ^a^Sex on substances was only measured at wave two and wave three.

**Supplementary Table 7. Univariable associations between hypothesised protective factors and HIV risk practices in girls and boys. N=1563, Observations=4402.**

|  | **Multiple sexual partners** | **Transactional sex** | **Age-disparate sex** | **Unprotected sex** | **Sex on substances^a^** |
| --- | --- | --- | --- | --- | --- |
|  | **OR (95%CI); p-value** | **OR (95%CI); p-value** | **OR (95%CI); p-value** | **OR (95%CI); p-value** | **OR (95%CI); p-value** |
| **Girls** |  |  |  |  |  |
| Number of social grants |  |  |  |  |  |
| Between | 0.88 (0.81-0.95); 0.002 | 0.86 (0.77-0.96); 0.008 | 0.82 (0.74-0.92); <.001 | 0.92 (0.86-0.98); 0.012 | 0.85 (0.74-0.96); 0.012 |
| Within | 0.93 (0.84-1.02); 0.134 | 0.93 (0.82-1.07); 0.325 | 0.94 (0.82-1.06); 0.316 | 0.96 (0.89-1.04); 0.304 | 0.81 (0.66-1.00); 0.051 |
| Positive caregiving |  |  |  |  |  |
| Between | 0.91 (0.89-0.94); <.001 | 0.88 (0.85-0.91); <.001 | 0.89 (0.86-0.92); <.001 | 0.93 (0.91-0.96); <.001 | 0.96 (0.92-1.00); 0.04 |
| Within | 0.97 (0.95-1.00); 0.043 | 0.97 (0.94-1.01); 0.153 | 0.97 (0.93-1.00); 0.047 | 0.98 (0.96-1.01); 0.126 | 1.00 (0.95-1.06); 0.983 |
| Caregiver supervision |  |  |  |  |  |
| Between | 0.86 (0.84-0.88); <.001 | 0.88 (0.85-0.90); <.001 | 0.89 (0.87-0.91); <.001 | 0.88 (0.87-0.90); <.001 | 0.85 (0.83-0.88); <.001 |
| Within | 1.01 (0.99-1.03); 0.181 | 0.97 (0.95-1.00); 0.026 | 0.99 (0.97-1.01); 0.394 | 1.01 (0.99-1.02); 0.426 | 0.98 (0.95-1.01); 0.238 |
| Adolescent-caregiver communication |  |  |  |  |  |
| Between | 0.91 (0.86-0.97); 0.002 | 1.00 (0.92-1.09); 0.979 | 0.92 (0.85-0.99); 0.02 | 0.96 (0.92-1.01); 0.092 | 0.90 (0.83-0.98); 0.017 |
| Within | 0.99 (0.94-1.03); 0.581 | 1.03 (0.97-1.11); 0.328 | 0.99 (0.93-1.05); 0.648 | 0.98 (0.94-1.02); 0.294 | 0.95 (0.87-1.04); 0.275 |
| Education enrolment |  |  |  |  |  |
| Between | 0.11 (0.08-0.15); <.001 | 0.13 (0.09-0.19); <.001 | 0.09 (0.06-0.13); <.001 | 0.11 (0.08-0.14); <.001 | 0.18 (0.11-0.28); <.001 |
| Within | 0.94 (0.59-1.50); 0.805 | 3.47 (1.82-6.60); <.001 | 0.32 (0.18-0.59); <.001 | 0.63 (0.42-0.93); 0.021 | 0.78 (0.29-2.11); 0.626 |
| Days with enough food |  |  |  |  |  |
| Between | 0.65 (0.58-0.74); <.001 | 0.60 (0.52-0.71); <.001 | 0.81 (0.69-0.96); 0.017 | 0.71 (0.64-0.79); <.001 | 0.82 (0.66-1.03); 0.092 |
| Within | 0.93 (0.82-1.05); 0.23 | 0.79 (0.68-0.92); 0.003 | 1.13 (0.95-1.35); 0.162 | 1.06 (0.95-1.18); 0.271 | 0.88 (0.66-1.18); 0.399 |
| **Boy** |  |  |  |  |  |
| Number of social grants |  |  |  |  |  |
| Between | 0.80 (0.73-0.88); <.001 | 0.88 (0.74-1.05); 0.166 | 0.83 (0.69-1.01); 0.057 | 0.89 (0.80-0.99); 0.026 | 0.86 (0.75-0.98); 0.02 |
| Within | 0.96 (0.85-1.07); 0.423 | 0.97 (0.78-1.20); 0.762 | 0.90 (0.71-1.13); 0.368 | 1.04 (0.91-1.18); 0.552 | 0.98 (0.81-1.20); 0.88 |
| Positive caregiving |  |  |  |  |  |
| Between | 0.92 (0.89-0.96); <.001 | 0.90 (0.84-0.97); 0.008 | 0.92 (0.85-1.00); 0.052 | 1.00 (0.96-1.05); 0.929 | 0.95 (0.90-1.00); 0.073 |
| Within | 0.98 (0.95-1.01); 0.125 | 0.92 (0.86-0.97); 0.004 | 0.97 (0.91-1.03); 0.317 | 1.03 (0.99-1.08); 0.093 | 1.00 (0.95-1.06); 0.889 |
| Caregiver supervision |  |  |  |  |  |
| Between | 0.86 (0.84-0.88); <.001 | 0.87 (0.84-0.90); <.001 | 0.88 (0.85-0.92); <.001 | 0.92 (0.90-0.94); <.001 | 0.86 (0.84-0.89); <.001 |
| Within | 0.97 (0.95-0.99); <.001 | 0.96 (0.93-0.99); 0.01 | 0.97 (0.93-1.00); 0.05 | 1.00 (0.98-1.02); 0.957 | 0.96 (0.92-1.00); 0.052 |
| Adolescent-caregiver communication |  |  |  |  |  |
| Between | 0.95 (0.89-1.02); 0.171 | 0.99 (0.86-1.14); 0.887 | 0.88 (0.76-1.01); 0.077 | 0.96 (0.92-1.01); 0.092 | 0.91 (0.83-1.00); 0.06 |
| Within | 1.02 (0.96-1.08); 0.511 | 1.00 (0.89-1.12); 0.953 | 1.02 (0.90-1.15); 0.752 | 0.98 (0.94-1.02); 0.294 | 0.96 (0.87-1.07); 0.485 |
| Education enrolment |  |  |  |  |  |
| Between | 0.05 (0.03-0.08); <.001 | 0.28 (0.09-0.83); 0.021 | 0.14 (0.05-0.36); <.001 | 0.13 (0.07-0.24); <.001 | 0.17 (0.09-0.31); <.001 |
| Within | 0.63 (0.33-1.20); 0.159 | 2.48 (0.61-10.06); 0.204 | 0.37 (0.10-1.34); 0.132 | 1.18 (0.54-2.54); 0.68 | 0.57 (0.16-1.95); 0.367 |
| Days with enough food |  |  |  |  |  |
| Between | 0.93 (0.77-1.11); 0.419 | 0.68 (0.52-0.90); 0.006 | 0.91 (0.63-1.31); 0.608 | 1.00 (0.80-1.25); 0.973 | 0.95 (0.72-1.24); 0.7 |
| Within | 1.01 (0.85-1.20); 0.912 | 0.86 (0.65-1.14); 0.285 | 1.08 (0.75-1.56); 0.689 | 0.95 (0.77-1.17); 0.638 | 1.06 (0.75-1.51); 0.733 |

^a^Sex on substances was only measured at wave two and wave three. Abbreviations: OR, odds ratio; CI, confidence interval.

**Supplementary Table 8. Intra-class correlations for HIV risk practices from null models (i.e. with no predictors).**

|  | **Variance** | **ICC (95%CI)** |
| --- | --- | --- |
|  | **Intercept** |  |
| **Girls** |  |  |
| Multiple sexual partners | 5.71 | 0.63 (0.56-0.70) |
| Transactional sex | 2.59 | 0.44 (0.32-0.57) |
| Age-disparate sex | 4.80 | 0.59 (0.48-0.70) |
| Condomless sex | 3.60 | 0.52 (0.45-0.59) |
| Sex on substances^a^ | 15.34 | 0.82 (0.66-0.92) |
| **Boy** |  |  |
| Multiple sexual partners | 6.51 | 0.66 (0.59-0.73) |
| Transactional sex | 0.81 | 0.20 (0.04-0.57) |
| Age-disparate sex | 2.56 | 0.44 (0.25-0.64) |
| Condomless sex | 1.65 | 0.33 (0.23-0.46) |
| Sex on substances^a^ | 9.69 | 0.75 (0.74-0.75) |

^a^Sex on substances was only measured at wave two and wave three.

Abbreviations: ICC, intra-class correlation; CI, confidence interval.

**Supplementary Table 9. Multivariable associations between hypothesised protective factors and HIV risk practices in girls and boys. Between- and within-individual effects are modelled separately for all protective factors. N=1563, Observations=2883.**

|  | **Multiple sexual partners** | | **Transactional sex** | | **Age-disparate sex** | | **Condomless sex** | | | **Sex on substances** | | |
| --- | --- | --- | --- | --- | --- | --- | --- | --- | --- | --- | --- | --- |
|  | **aOR (95%CI)** | **p-value^a^** | **aOR (95%CI)** | **p-value^a^** | **aOR (95%CI)** | **p-value^a^** | **aOR (95%CI)** | **p-value^a^** | **aOR (95%CI)** | | **p-value^a^** |  |
| **Girls** |  |  |  |  |  |  |  |  |  | |  |  |
| Number of social grants |  |  |  |  |  |  |  |  |  | |  |  |
| Between | 0.99 (0.84-1.18) | 0.906 | 0.98 (0.82-1.18) | 0.984 | 0.96 (0.79-1.16) | 0.091 | 1.08 (0.96-1.23) | 0.927 | 0.95 (0.73-1.23) | | 0.172 |  |
| Within | 0.98 (0.85-1.14) |  | 0.98 (0.82-1.17) |  | 1.17 (0.97-1.41) |  | 1.08 (0.96-1.20) |  | 0.75 (0.56-1.01) | |  |  |
| Positive caregiving |  |  |  |  |  |  |  |  |  | |  |  |
| Between | 1.03 (0.97-1.09) | 0.093 | 0.94 (0.89-1.00) | 0.999 | 0.95 (0.89-1.02) | 0.753 | 1.03 (0.98-1.08) | 0.151 | 1.04 (0.96-1.12) | | 0.830 |  |
| Within | 0.97 (0.93-1.00) |  | 0.94 (0.90-0.99) |  | 0.97 (0.92-1.01) |  | 0.99 (0.96-1.02) |  | 1.05 (0.97-1.14) | |  |  |
| Caregiver supervision |  |  |  |  |  |  |  |  |  | |  |  |
| Between | 0.90 (0.86-0.94) | **<.001** | 0.92 (0.89-0.96) | 0.184 | 0.94 (0.90-0.99) | 0.169 | 0.94 (0.92-0.97) | **<.001** | 0.86 (0.81-0.92) | | **0.002** |  |
| Within | 1.02 (0.99-1.04) |  | 0.96 (0.93-0.99) |  | 0.98 (0.95-1.01) |  | 1.01 (0.99-1.03) |  | 0.98 (0.93-1.02) | |  |  |
| Adolescent-caregiver communication |  |  |  |  |  |  |  |  |  | |  |  |
| Between | 0.94 (0.85-1.05) | 0.306 | 1.12 (1.00-1.26) | 0.657 | 0.91 (0.81-1.03) | 0.235 | 0.99 (0.92-1.08) | 0.607 | 0.94 (0.81-1.10) | | 0.660 |  |
| Within | 1.00 (0.94-1.07) |  | 1.09 (1.00-1.18) |  | 1.00 (0.92-1.08) |  | 0.97 (0.92-1.02) |  | 0.90 (0.78-1.04) | |  |  |
| Education enrolment |  |  |  |  |  |  |  |  |  | |  |  |
| Between | 0.82 (0.41-1.65) | 0.292 | 1.26 (0.61-2.58) | 0.496 | 0.33 (0.15-0.72) | 0.380 | 0.40 (0.23-0.70) | **0.023** | 0.92 (0.30-2.75) | | 0.881 |  |
| Within | 1.34 (0.74-2.43) |  | 1.79 (0.84-3.84) |  | 0.53 (0.25-1.10) |  | 0.95 (0.58-1.56) |  | 0.81 (0.27-2.42) | |  |  |
| Days with enough food |  |  |  |  |  |  |  |  |  | |  |  |
| Between | 0.78 (0.61-1.00) | 0.564 | 0.76 (0.60-0.98) | 0.811 | 1.23 (0.91-1.66) | 0.675 | 0.83 (0.69-1.01) | **0.023** | 0.85 (0.56-1.28) | | 0.640 |  |
| Within | 0.85 (0.72-1.01) |  | 0.80 (0.66-0.97) |  | 1.13 (0.91-1.42) |  | 1.09 (0.95-1.26) |  | 0.95 (0.69-1.32) | |  |  |
| HIV status - Living with HIV | 1.15 (0.73-1.82) |  | 1.10 (0.67-1.82) |  | 1.16 (0.68-1.98) |  | 0.60 (0.43-0.84) |  | 0.65 (0.31-1.36) | |  |  |
| Rural location - Yes | 1.26 (0.81-1.96) |  | 0.92 (0.56-1.50) |  | 0.78 (0.46-1.33) |  | 0.95 (0.68-1.33) |  | 0.88 (0.41-1.91) | |  |  |
| Informal housing - Yes | 0.85 (0.50-1.44) |  | 1.32 (0.77-2.26) |  | 1.13 (0.62-2.05) |  | 1.10 (0.74-1.63) |  | 0.60 (0.22-1.64) | |  |  |
| Household size | 1.01 (0.94-1.09) |  | 1.02 (0.93-1.11) |  | 0.97 (0.88-1.07) |  | 0.96 (0.90-1.01) |  | 1.03 (0.90-1.18) | |  |  |
| Maternal orphan - Yes | 1.14 (0.70-1.86) |  | 0.84 (0.50-1.40) |  | 0.70 (0.38-1.28) |  | 0.84 (0.58-1.20) |  | 0.61 (0.26-1.46) | |  |  |
| Paternal orphan - Yes | 0.89 (0.58-1.36) |  | 2.14 (1.34-3.42) |  | 0.96 (0.56-1.66) |  | 1.11 (0.81-1.53) |  | 1.51 (0.70-3.24) | |  |  |
| Age | 1.72 (1.53-1.94) |  | 1.58 (1.39-1.79) |  | 1.46 (1.29-1.66) |  | 1.40 (1.30-1.50) |  | 1.52 (1.26-1.83) | |  |  |
| Wave | 0.69 (0.53-0.88) |  | 0.30 (0.21-0.42) |  | 1.02 (0.75-1.37) |  | 0.95 (0.79-1.14) |  | 0.72 (0.39-1.33) | |  |  |
| Variance components |  |  |  |  |  |  |  |  |  | |  |  |
| Level 2: In level-1 intercept | 2.52 |  | 1.15 |  | 2.20 |  | 1.51 |  | 3.65 | |  |  |
| Goodness of fit |  |  |  |  |  |  |  |  |  | |  |  |
| AUC | 0.96 |  | 0.94 |  | 0.96 |  | 0.93 |  | 0.99 | |  |  |
| **Boy** |  |  |  |  |  |  |  |  |  | |  |  |
| Number of social grants |  |  |  |  |  |  |  |  |  | |  |  |
| Between | 1.06 (0.87-1.29) | 0.923 | 1.04 (0.84-1.30) | 0.774 | 0.98 (0.75-1.28) | 0.720 | 1.07 (0.93-1.24) | 0.838 | 1.01 (0.79-1.29) | | 0.674 |  |
| Within | 1.04 (0.89-1.23) |  | 1.00 (0.79-1.26) |  | 0.92 (0.70-1.20) |  | 1.09 (0.95-1.26) |  | 1.09 (0.83-1.42) | |  |  |
| Positive caregiving |  |  |  |  |  |  |  |  |  | |  |  |
| Between | 1.02 (0.93-1.11) | 0.722 | 1.01 (0.91-1.10) | 0.110 | 1.03 (0.91-1.16) | 0.683 | 1.05 (0.99-1.12) | 0.635 | 1.06 (0.95-1.18) | | 0.897 |  |
| Within | 1.00 (0.95-1.05) |  | 0.91 (0.84-0.97) |  | 1.00 (0.92-1.08) |  | 1.07 (1.02-1.12) |  | 1.05 (0.97-1.14) | |  |  |
| Caregiver supervision |  |  |  |  |  |  |  |  |  | |  |  |
| Between | 0.91 (0.86-0.96) | 0.430 | 0.91 (0.86-0.96) | 0.255 | 0.93 (0.87-1.00) | 0.785 | 0.98 (0.94-1.01) | 0.455 | 0.89 (0.83-0.95) | | 0.178 |  |
| Within | 0.93 (0.90-0.96) |  | 0.95 (0.91-0.98) |  | 0.95 (0.90-0.99) |  | 0.99 (0.97-1.02) |  | 0.94 (0.89-0.99) | |  |  |
| Adolescent-caregiver communication |  |  |  |  |  |  |  |  |  | |  |  |
| Between | 1.04 (0.90-1.19) | 0.983 | 1.02 (0.88-1.18) | 0.961 | 0.89 (0.73-1.09) | 0.313 | 0.96 (0.87-1.07) | 0.943 | 0.97 (0.82-1.15) | | 0.644 |  |
| Within | 1.04 (0.95-1.13) |  | 1.01 (0.90-1.14) |  | 1.01 (0.88-1.15) |  | 0.96 (0.89-1.04) |  | 0.92 (0.79-1.07) | |  |  |
| Education enrolment |  |  |  |  |  |  |  |  |  | |  |  |
| Between | 1.16 (0.33-4.06) | 0.443 | 6.76 (1.50-30.39) | 0.925 | 1.99 (0.34-11.53) | 0.764 | 0.73 (0.29-1.87) | **0.044** | 3.72 (0.60-22.94) | | 0.174 |  |
| Within | 2.13 (0.85-5.30) |  | 6.18 (1.50-25.38) |  | 1.39 (0.35-5.46) |  | 2.70 (1.14-6.38) |  | 0.64 (0.16-2.54) | |  |  |
| Days with enough food |  |  |  |  |  |  |  |  |  | |  |  |
| Between | 1.03 (0.70-1.50) | 0.992 | 0.68 (0.49-0.95) | 0.370 | 1.00 (0.60-1.65) | 0.757 | 1.12 (0.85-1.48) | 0.253 | 1.00 (0.59-1.72) | | 0.842 |  |
| Within | 1.03 (0.78-1.35) |  | 0.85 (0.62-1.17) |  | 1.11 (0.70-1.76) |  | 0.90 (0.70-1.15) |  | 1.08 (0.70-1.66) | |  |  |
| HIV status - Living with HIV | 0.56 (0.32-0.99) |  | 1.26 (0.67-2.35) |  | 1.28 (0.57-2.86) |  | 0.56 (0.38-0.84) |  | 0.28 (0.12-0.64) | |  |  |
| Rural location - Yes | 0.77 (0.41-1.47) |  | 1.31 (0.66-2.60) |  | 0.60 (0.21-1.67) |  | 0.48 (0.28-0.81) |  | 0.54 (0.20-1.45) | |  |  |
| Informal housing - Yes | 0.53 (0.22-1.29) |  | 0.93 (0.37-2.37) |  | 0.42 (0.09-1.95) |  | 0.96 (0.51-1.79) |  | 0.25 (0.05-1.31) | |  |  |
| Household size | 0.94 (0.86-1.04) |  | 1.01 (0.91-1.13) |  | 1.03 (0.90-1.18) |  | 0.98 (0.91-1.06) |  | 1.01 (0.89-1.15) | |  |  |
| Maternal orphan - Yes | 0.87 (0.48-1.56) |  | 0.78 (0.39-1.54) |  | 0.61 (0.24-1.53) |  | 0.58 (0.37-0.92) |  | 0.94 (0.37-2.35) | |  |  |
| Paternal orphan - Yes | 0.71 (0.43-1.17) |  | 0.87 (0.45-1.68) |  | 0.75 (0.33-1.69) |  | 1.06 (0.69-1.62) |  | 1.39 (0.63-3.06) | |  |  |
| Age | 2.16 (1.86-2.50) |  | 1.51 (1.33-1.71) |  | 1.57 (1.32-1.86) |  | 1.36 (1.25-1.49) |  | 1.78 (1.45-2.19) | |  |  |
| Wave | 0.90 (0.67-1.22) |  | 0.80 (0.53-1.20) |  | 1.31 (0.80-2.14) |  | 1.35 (1.05-1.74) |  | 0.65 (0.34-1.22) | |  |  |
| Variance components |  |  |  |  |  |  |  |  |  | |  |  |
| Level 2: In level-1 intercept | 2.69 |  | 0.00 |  | 1.71 |  | 0.64 |  | 3.38 | |  |  |
| Goodness of fit |  |  |  |  |  |  |  |  |  | |  |  |
| AUC | 0.97 |  | 0.87 |  | 0.96 |  | 0.88 |  | 0.98 | |  |  |

^a^Wald p-value for equality across pairs of between- and within-individual coefficients. A significant p-value indicates that pairs of coefficients are different from one another. The AUC statistic ranges from 0 to 1 and gives us an idea of how well a model is able to distinguish between positive and negative outcomes. The higher the AUC, the better the model is at correctly classifying outcomes.

Abbreviations: aOR, adjusted odds ratio; CI, confidence interval; signif. significance; equal. Equality; AUC, Area Under the Curve.

**Supplementary Table 10. Summary of adjusted odds ratios for additional covariates included in models investigating multivariable associations between hypothesised protective factors and HIV risk practices in girls and boys.**

|  | **Multiple sexual partners** | **Transactional sex** | **Age-disparate sex** | **Condomless sex** | **Sex on substances** |
| --- | --- | --- | --- | --- | --- |
|  | **aOR (95%CI); p-value** | **aOR (95%CI); p-value** | **aOR (95%CI); p-value** | **aOR (95%CI); p-value** | **aOR (95%CI); p-value** |
| **Girls** |  |  |  |  |  |
| HIV status - Living with HIV | 1.18 (0.75-1.85); 0.481 | 1.13 (0.69-1.84); 0.632 | 1.18 (0.70-1.97); 0.530 | 0.61 (0.44-0.85); 0.004 | 0.60 (0.30-1.23); 0.163 |
| Rural location - Yes | 1.22 (0.79-1.89); 0.379 | 0.91 (0.56-1.48); 0.710 | 0.78 (0.46-1.31); 0.342 | 0.92 (0.66-1.29); 0.632 | 0.92 (0.43-1.95); 0.832 |
| Informal housing - Yes | 0.87 (0.52-1.46); 0.597 | 1.40 (0.83-2.37); 0.211 | 1.18 (0.66-2.11); 0.583 | 1.11 (0.75-1.65); 0.586 | 0.55 (0.21-1.47); 0.234 |
| Household size | 1.01 (0.94-1.09); 0.731 | 1.02 (0.93-1.11); 0.700 | 0.96 (0.88-1.06); 0.428 | 0.96 (0.90-1.01); 0.146 | 1.04 (0.91-1.19); 0.539 |
| Maternal orphan - Yes | 1.10 (0.68-1.79); 0.687 | 0.81 (0.49-1.35); 0.427 | 0.69 (0.38-1.26); 0.227 | 0.82 (0.57-1.18); 0.295 | 0.66 (0.28-1.53); 0.328 |
| Paternal orphan - Yes | 0.90 (0.59-1.36); 0.607 | 2.12 (1.33-3.38); 0.002 | 0.95 (0.55-1.62); 0.840 | 1.12 (0.81-1.54); 0.504 | 1.55 (0.73-3.29); 0.255 |
| Age | 1.74 (1.56-1.95); 0.000 | 1.64 (1.45-1.84); 0.000 | 1.53 (1.36-1.72); 0.000 | 1.39 (1.29-1.50); 0.000 | 1.50 (1.26-1.78); 0.000 |
| Wave | 0.67 (0.53-0.84); 0.001 | 0.28 (0.20-0.38); 0.000 | 0.92 (0.70-1.20); 0.522 | 0.96 (0.80-1.15); 0.642 | 0.71 (0.40-1.25); 0.233 |
| **Boys** |  |  |  |  |  |
| HIV status - Living with HIV | 0.54 (0.31-0.96); 0.036 | 1.22 (0.66-2.28); 0.524 | 1.27 (0.57-2.82); 0.555 | 0.56 (0.38-0.84); 0.005 | 0.29 (0.13-0.66); 0.003 |
| Rural location - Yes | 0.74 (0.40-1.38); 0.340 | 1.14 (0.59-2.21); 0.705 | 0.56 (0.21-1.54); 0.263 | 0.48 (0.28-0.80); 0.005 | 0.52 (0.20-1.35); 0.179 |
| Informal housing - Yes | 0.52 (0.21-1.26); 0.147 | 0.90 (0.35-2.28); 0.819 | 0.43 (0.09-1.96); 0.273 | 0.95 (0.51-1.78); 0.869 | 0.27 (0.05-1.37); 0.113 |
| Household size | 0.94 (0.86-1.03); 0.215 | 1.03 (0.93-1.14); 0.634 | 1.04 (0.91-1.18); 0.566 | 0.98 (0.91-1.05); 0.563 | 1.00 (0.88-1.14); 0.965 |
| Maternal orphan - Yes | 0.86 (0.48-1.54); 0.620 | 0.74 (0.38-1.44); 0.371 | 0.58 (0.24-1.45); 0.245 | 0.60 (0.38-0.94); 0.027 | 0.98 (0.40-2.38); 0.958 |
| Paternal orphan - Yes | 0.72 (0.44-1.18); 0.195 | 0.92 (0.48-1.76); 0.807 | 0.77 (0.34-1.73); 0.525 | 1.04 (0.68-1.59); 0.847 | 1.25 (0.58-2.70); 0.571 |
| Age | 2.21 (1.92-2.55); 0.000 | 1.51 (1.34-1.70); 0.000 | 1.55 (1.33-1.82); 0.000 | 1.38 (1.27-1.50); 0.000 | 1.77 (1.47-2.14); 0.000 |
| Wave | 0.85 (0.65-1.11); 0.231 | 0.82 (0.56-1.19); 0.290 | 1.34 (0.86-2.10); 0.200 | 1.31 (1.03-1.68); 0.030 | 0.68 (0.38-1.24); 0.208 |

^a^Sex on substances was only measured at wave two and wave three.

Abbreviations: aOR, adjusted odds ratio; CI, confidence interval.

**Supplementary Table 11. Multivariable lagged associations between hypothesised protective factors and HIV risk practices in girls and boys. Between- and within-individual effects are modelled separately for all protective factors. N=1563, Observations=2883.**

|  | **Multiple sexual partners** | | **Transactional sex** | | **Age-disparate sex** | | **Condomless sex** | | **Sex on substances** | |
| --- | --- | --- | --- | --- | --- | --- | --- | --- | --- | --- |
|  | **aOR (95%CI)** | **p-value^a^** | **aOR (95%CI)** | **p-value^a^** | **aOR (95%CI)** | **p-value^a^** | **aOR (95%CI)** | **p-value^a^** | **aOR (95%CI)** | **p-value^a^** |
| **Girls** |  |  |  |  |  |  |  |  |  |  |
| Number of social grants |  |  |  |  |  |  |  |  |  |  |
| Between | 1.10 (0.92-1.32) | 0.612 | 1.05 (0.83-1.32) | 0.102 | 0.99 (0.82-1.21) | 0.370 | 1.11 (0.92-1.32) | 0.109 | 0.94 (0.73-1.20) | 0.458 |
| Within | 1.02 (0.83-1.25) |  | 1.44 (1.07-1.95) |  | 0.86 (0.68-1.09) |  | 0.93 (0.83-1.25) |  | 1.08 (0.82-1.44) |  |
| Positive caregiving |  |  |  |  |  |  |  |  |  |  |
| Between | 1.02 (0.96-1.09) | 0.852 | 0.91 (0.85-0.99) | 0.123 | 0.96 (0.90-1.03) | 0.170 | 1.01 (0.96-1.09) | 0.604 | 1.02 (0.94-1.11) | 0.858 |
| Within | 1.03 (0.97-1.08) |  | 0.99 (0.92-1.07) |  | 1.02 (0.96-1.09) |  | 1.03 (0.97-1.08) |  | 1.01 (0.93-1.09) |  |
| Caregiver supervision |  |  |  |  |  |  |  |  |  |  |
| Between | 0.93 (0.89-0.97) | 0.127 | 0.95 (0.90-1.00) | **0.028** | 0.94 (0.90-0.99) | **0.004** | 0.94 (0.89-0.97) | 0.179 | 0.91 (0.85-0.96) | **0.010** |
| Within | 0.97 (0.94-1.01) |  | 1.03 (0.97-1.08) |  | 1.03 (0.99-1.08) |  | 0.97 (0.94-1.01) |  | 1.00 (0.95-1.05) |  |
| Adolescent-caregiver communication |  |  |  |  |  |  |  |  |  |  |
| Between | 0.93 (0.83-1.05) | 0.416 | 1.11 (0.95-1.29) | **0.030** | 0.92 (0.82-1.04) | 0.290 | 1.05 (0.83-1.05) | 0.231 | 0.96 (0.82-1.12) | 0.307 |
| Within | 0.99 (0.90-1.09) |  | 0.87 (0.75-1.01) |  | 1.01 (0.90-1.13) |  | 0.98 (0.90-1.09) |  | 1.07 (0.93-1.22) |  |
| Education enrolment |  |  |  |  |  |  |  |  |  |  |
| Between | 1.08 (0.50-2.35) | 0.115 | 1.19 (0.47-3.06) | 0.327 | 0.58 (0.26-1.30) | 0.941 | 0.66 (0.50-2.35) | 0.425 | 0.60 (0.22-1.67) | 0.602 |
| Within | 3.16 (1.04-9.55) |  | 0.49 (0.10-2.28) |  | 0.61 (0.18-2.09) |  | 1.02 (1.04-9.55) |  | 0.96 (0.23-4.05) |  |
| Days with enough food |  |  |  |  |  |  |  |  |  |  |
| Between | 0.87 (0.68-1.11) | 0.586 | 0.83 (0.63-1.10) | 0.082 | 1.04 (0.80-1.36) | 0.166 | 0.84 (0.68-1.11) | 0.687 | 0.95 (0.68-1.33) | 0.579 |
| Within | 0.79 (0.62-1.00) |  | 1.20 (0.87-1.64) |  | 0.79 (0.59-1.05) |  | 0.80 (0.62-1.00) |  | 1.09 (0.77-1.53) |  |
| HIV status - Living with HIV | 1.08 (0.62-1.89) |  | 1.75 (0.83-3.69) |  | 0.96 (0.53-1.73) |  | 0.55 (0.62-1.89) |  | 0.58 (0.28-1.23) |  |
| Rural location - Yes | 1.12 (0.64-1.96) |  | 1.53 (0.78-3.01) |  | 0.79 (0.43-1.46) |  | 0.95 (0.64-1.96) |  | 1.16 (0.53-2.53) |  |
| Informal housing - Yes | 0.81 (0.40-1.64) |  | 0.96 (0.40-2.31) |  | 1.21 (0.59-2.48) |  | 0.91 (0.40-1.64) |  | 0.57 (0.21-1.57) |  |
| Household size | 0.99 (0.91-1.08) |  | 1.05 (0.94-1.18) |  | 1.02 (0.92-1.12) |  | 0.96 (0.91-1.08) |  | 1.01 (0.90-1.14) |  |
| Maternal orphan - Yes | 1.36 (0.72-2.54) |  | 0.57 (0.26-1.23) |  | 0.91 (0.44-1.89) |  | 0.83 (0.72-2.54) |  | 0.84 (0.35-2.02) |  |
| Paternal orphan - Yes | 0.69 (0.40-1.20) |  | 4.29 (2.02-9.10) |  | 0.72 (0.36-1.44) |  | 1.06 (0.40-1.20) |  | 1.41 (0.65-3.05) |  |
| Age | 1.79 (1.55-2.06) |  | 1.45 (1.23-1.71) |  | 1.51 (1.31-1.74) |  | 1.36 (1.55-2.06) |  | 1.58 (1.31-1.90) |  |
| Wave | 0.64 (0.41-1.00) |  | 0.33 (0.16-0.65) |  | 0.82 (0.49-1.39) |  | 0.75 (0.41-1.00) |  | 0.69 (0.37-1.29) |  |
| Variance components |  |  |  |  |  |  |  |  |  |  |
| Level 2: In level-1 intercept | 3.02 |  | 1.53 |  | 2.13 |  | 1.77 |  | 3.67 |  |
| Goodness of fit |  |  |  |  |  |  |  |  |  |  |
| AUC | 0.97 |  | 0.95 |  | 0.97 |  | 0.94 |  | 0.99 |  |
| **Boy** |  |  |  |  |  |  |  |  |  |  |
| Number of social grants |  |  |  |  |  |  |  |  |  |  |
| Between | 1.06 (0.83-1.35) | 0.958 | 1.11 (0.87-1.44) | 0.168 | 1.01 (0.78-1.33) | 0.936 | 1.01 (0.83-1.35) | 0.517 | 0.97 (0.73-1.29) | 0.815 |
| Within | 1.05 (0.81-1.35) |  | 0.83 (0.60-1.15) |  | 1.00 (0.71-1.40) |  | 0.92 (0.81-1.35) |  | 1.02 (0.74-1.40) |  |
| Positive caregiving |  |  |  |  |  |  |  |  |  |  |
| Between | 1.05 (0.96-1.15) | 0.136 | 1.00 (0.92-1.09) | 0.914 | 1.03 (0.94-1.14) | 0.160 | 1.02 (0.96-1.15) | 0.170 | 1.05 (0.94-1.17) | 0.038 |
| Within | 0.96 (0.89-1.03) |  | 0.99 (0.90-1.09) |  | 0.93 (0.83-1.03) |  | 0.96 (0.89-1.03) |  | 0.89 (0.81-0.99) |  |
| Caregiver supervision |  |  |  |  |  |  |  |  |  |  |
| Between | 0.93 (0.88-0.99) | **0.006** | 0.94 (0.89-0.99) | **<.001** | 0.97 (0.91-1.03) | 0.156 | 0.99 (0.88-0.99) | 0.308 | 0.91 (0.86-0.98) | **0.004** |
| Within | 1.03 (0.98-1.07) |  | 1.07 (1.01-1.13) |  | 1.03 (0.97-1.09) |  | 0.97 (0.98-1.07) |  | 1.03 (0.98-1.08) |  |
| Adolescent-caregiver communication |  |  |  |  |  |  |  |  |  |  |
| Between | 1.11 (0.95-1.29) | 0.470 | 1.12 (0.97-1.30) | 0.372 | 0.93 (0.79-1.09) | 0.609 | 0.98 (0.95-1.29) | 0.305 | 1.03 (0.86-1.22) | 0.192 |
| Within | 1.03 (0.91-1.17) |  | 1.01 (0.86-1.20) |  | 0.99 (0.83-1.17) |  | 1.05 (0.91-1.17) |  | 1.20 (1.02-1.42) |  |
| Education enrolment |  |  |  |  |  |  |  |  |  |  |
| Days with enough food |  |  |  |  |  |  |  |  |  |  |
| Between | 0.99 (0.67-1.48) | 0.511 | 0.69 (0.50-0.95) | **0.021** | 1.01 (0.66-1.54) | 0.873 | 1.08 (0.67-1.48) | 0.171 | 0.92 (0.57-1.46) | 0.143 |
| Within | 1.18 (0.84-1.64) |  | 1.20 (0.81-1.76) |  | 1.06 (0.66-1.70) |  | 0.82 (0.84-1.64) |  | 1.43 (0.93-2.20) |  |
| HIV status - Living with HIV | 0.66 (0.32-1.36) |  | 1.27 (0.60-2.65) |  | 1.24 (0.55-2.80) |  | 0.67 (0.32-1.36) |  | 0.27 (0.11-0.66) |  |
| Rural location - Yes | 1.25 (0.56-2.76) |  | 1.24 (0.57-2.73) |  | 0.58 (0.21-1.64) |  | 0.49 (0.56-2.76) |  | 0.62 (0.22-1.74) |  |
| Informal housing - Yes | 0.63 (0.20-1.97) |  | 1.28 (0.46-3.54) |  | 0.32 (0.06-1.74) |  | 0.98 (0.20-1.97) |  | 0.15 (0.02-1.07) |  |
| Household size | 0.93 (0.83-1.04) |  | 1.00 (0.89-1.13) |  | 1.02 (0.90-1.16) |  | 1.01 (0.83-1.04) |  | 1.01 (0.88-1.16) |  |
| Maternal orphan - Yes | 0.85 (0.40-1.81) |  | 0.86 (0.41-1.81) |  | 0.63 (0.26-1.53) |  | 0.51 (0.40-1.81) |  | 1.12 (0.43-2.91) |  |
| Paternal orphan - Yes | 0.71 (0.38-1.32) |  | 0.87 (0.44-1.73) |  | 0.78 (0.35-1.71) |  | 1.14 (0.38-1.32) |  | 1.42 (0.63-3.19) |  |
| Age | 2.36 (1.93-2.88) |  | 1.51 (1.30-1.76) |  | 1.53 (1.30-1.81) |  | 1.31 (1.93-2.88) |  | 1.87 (1.51-2.31) |  |
| Wave | 0.57 (0.35-0.93) |  | 0.20 (0.09-0.42) |  | 0.48 (0.24-0.94) |  | 0.46 (0.35-0.93) |  | 0.68 (0.37-1.25) |  |
| Variance components |  |  |  |  |  |  |  |  |  |  |
| Level 2: In level-1 intercept | 4.29 |  | 0.31 |  | 1.37 |  | 0.60 |  | 3.79 |  |
| Goodness of fit |  |  |  |  |  |  |  |  |  |  |
| AUC | 0.98 |  | 0.89 |  | 0.95 |  | 0.85 |  | 0.99 |  |

^a^Wald p-value for equality across pairs of between- and within-individual coefficients. A significant p-value indicates that pairs of coefficients are different from one another. ^b^Wald p-value for significance. A significant p-value indicates that a coefficient is significantly different from 1.00.

Abbreviations: aOR, adjusted odds ratio; CI, confidence interval; signif. significance; equal. Equality; AUC, Area Under the Curve.

**Supplementary Table 12. Multivariable lagged associations between hypothesised protective factors and HIV risk practices in girls and boys. Average effects are modelled when there is no evidence that within- and between-individual effects are different. N=1563, Observations=2883.**

|  | **Multiple sexual partners** | | | **Transactional sex** | | | **Age-disparate sex** | | | | **Condomless sex** | | | **Sex on substances** | | |
| --- | --- | --- | --- | --- | --- | --- | --- | --- | --- | --- | --- | --- | --- | --- | --- | --- |
|  | **aOR (95%CI)** | **naïve**  **p-value^a^** | **sharpened q-value** | **aOR (95%CI)** | **naïve**  **p-value^a^** | **sharpened q-value** | **aOR (95%CI)** | **naïve**  **p-value^a^** | **sharpened q-value** | **aOR (95%CI)** | | **naïve**  **p-value^a^** | **sharpened q-value** | **aOR (95%CI)** | **naïve**  **p-value^a^** | **sharpened q-value** |
| **Girls** |  |  |  |  |  |  |  |  |  |  | |  |  |  |  |  |
| Number of social grants |  |  |  |  |  |  |  |  |  |  | |  |  |  |  |  |
| Between |  |  |  |  |  |  |  |  |  |  | |  |  |  |  |  |
| Within |  |  |  |  |  |  |  |  |  |  | |  |  |  |  |  |
| Average | 1.06 (0.93-1.21) | 0.353 | 0.319 | 1.17 (0.97-1.41) | 0.095 | 0.247 | 0.94 (0.81-1.09) | 0.396 | 0.822 | 1.03 (0.94-1.14) | | 0.526 | 0.422 | 1.00 (0.83-1.20) | 0.981 | 1.000 |
| Positive caregiving |  |  |  |  |  |  |  |  |  |  | |  |  |  |  |  |
| Between |  |  |  |  |  |  |  |  |  |  | |  |  |  |  |  |
| Within |  |  |  |  |  |  |  |  |  |  | |  |  |  |  |  |
| Average | 1.03 (0.98-1.07) | 0.244 | 0.319 | 0.95 (0.90-1.00) | 0.072 | 0.247 | 0.99 (0.95-1.04) | 0.775 | 1.000 | 1.03 (0.99-1.06) | | 0.115 | 0.182 | 1.01 (0.96-1.07) | 0.663 | 1.000 |
| Caregiver supervision |  |  |  |  |  |  |  |  |  |  | |  |  |  |  |  |
| Between |  |  |  | 0.94 (0.89-0.99) | 0.026 | 0.247 | 0.95 (0.91-0.99) | 0.017 | 0.135 |  | |  |  | **0.90 (0.85-0.96)** | **<.001** | **0.004** |
| Within |  |  |  | 1.03 (0.98-1.08) | 0.231 | 0.247 | 1.02 (0.98-1.07) | 0.260 | 0.822 |  | |  |  | 1.00 (0.96-1.05) | 0.947 | 1.000 |
| Average | **0.95 (0.93-0.98)** | **<.001** | **0.003** |  |  |  |  |  |  | **0.96 (0.94-0.98)** | | **<.001** | **0.002** |  |  |  |
| Adolescent-caregiver communication |  |  |  |  |  |  |  |  |  |  | |  |  |  |  |  |
| Between |  |  |  | 1.08 (0.93-1.26) | 0.289 | 0.247 |  |  |  |  | |  |  |  |  |  |
| Within |  |  |  | 0.88 (0.76-1.02) | 0.099 | 0.247 |  |  |  |  | |  |  |  |  |  |
| Average | 0.97 (0.90-1.04) | 0.362 | 0.319 |  |  |  | 0.97 (0.90-1.05) | 0.485 | 0.822 | 1.01 (0.96-1.07) | | 0.678 | 0.422 | 1.02 (0.92-1.13) | 0.693 | 1.000 |
| Education enrolment |  |  |  |  |  |  |  |  |  |  | |  |  |  |  |  |
| Between |  |  |  |  |  |  |  |  |  |  | |  |  |  |  |  |
| Within |  |  |  |  |  |  |  |  |  |  | |  |  |  |  |  |
| Average | 1.54 (0.82-2.89) | 0.180 | 0.315 | 0.84 (0.38-1.89) | 0.681 | 0.588 | 0.60 (0.31-1.16) | 0.129 | 0.631 | 0.72 (0.44-1.19) | | 0.198 | 0.247 | 0.72 (0.32-1.63) | 0.429 | 1.000 |
| Days with enough food |  |  |  |  |  |  |  |  |  |  | |  |  |  |  |  |
| Between |  |  |  |  |  |  |  |  |  |  | |  |  |  |  |  |
| Within |  |  |  |  |  |  |  |  |  |  | |  |  |  |  |  |
| Average | **0.81 (0.69-0.96)** | **0.015** | **0.039** | 0.98 (0.79-1.20) | 0.823 | 0.588 | 0.91 (0.75-1.10) | 0.325 | 0.822 | **0.82 (0.72-0.93)** | | **0.002** | **0.006** | 1.01 (0.79-1.29) | 0.938 | 1.000 |
| HIV status - Living with HIV | 1.09 (0.63-1.88) | 0.754 |  | 1.75 (0.82-3.71) | 0.147 |  | 0.91 (0.51-1.63) | 0.762 |  | 0.56 (0.38-0.83) | | 0.004 |  | 0.59 (0.28-1.24) | 0.164 |  |
| Rural location - Yes | 1.15 (0.66-1.97) | 0.625 |  | 1.57 (0.80-3.11) | 0.191 |  | 0.86 (0.48-1.57) | 0.634 |  | 0.97 (0.65-1.45) | | 0.893 |  | 1.15 (0.53-2.48) | 0.717 |  |
| Informal housing - Yes | 0.89 (0.45-1.75) | 0.732 |  | 0.95 (0.40-2.28) | 0.909 |  | 1.19 (0.59-2.42) | 0.621 |  | 0.94 (0.57-1.55) | | 0.819 |  | 0.56 (0.21-1.54) | 0.261 |  |
| Household size | 1.00 (0.92-1.08) | 0.922 |  | 1.01 (0.91-1.13) | 0.834 |  | 1.03 (0.94-1.12) | 0.543 |  | 0.98 (0.92-1.04) | | 0.558 |  | 0.99 (0.89-1.11) | 0.918 |  |
| Maternal orphan - Yes | 1.29 (0.70-2.37) | 0.414 |  | 0.58 (0.27-1.29) | 0.182 |  | 0.93 (0.46-1.90) | 0.840 |  | 0.82 (0.53-1.28) | | 0.388 |  | 0.82 (0.35-1.95) | 0.660 |  |
| Paternal orphan - Yes | 0.71 (0.41-1.22) | 0.218 |  | 4.25 (1.98-9.09) | <.001 |  | 0.76 (0.39-1.49) | 0.427 |  | 1.06 (0.71-1.58) | | 0.771 |  | 1.44 (0.67-3.08) | 0.353 |  |
| Age | 1.87 (1.62-2.15) | <.001 |  | 1.46 (1.24-1.71) | <.001 |  | 1.52 (1.33-1.75) | <.001 |  | 1.38 (1.28-1.50) | | <.001 |  | 1.61 (1.34-1.93) | <.001 |  |
| Wave | 0.56 (0.37-0.85) | 0.006 |  | 0.32 (0.17-0.63) | <.001 |  | 0.76 (0.47-1.23) | 0.265 |  | 0.74 (0.54-1.01) | | 0.055 |  | 0.62 (0.35-1.11) | 0.109 |  |
| Variance components |  |  |  |  |  |  |  |  |  |  | |  |  |  |  |  |
| Level 2: In level-1 intercept | 2.90 |  |  | 1.75 |  |  | 2.08 |  |  | 1.75 | |  |  | 3.69 |  |  |
| Goodness of fit |  |  |  |  |  |  |  |  |  |  | |  |  |  |  |  |
| AUC | 0.97 |  |  | 0.95 |  |  | 0.97 |  |  | 0.94 | |  |  | 0.99 |  |  |
| **Boys** |  |  |  |  |  |  |  |  |  |  | |  |  |  |  |  |
| Number of social grants |  |  |  |  |  |  |  |  |  |  | |  |  |  |  |  |
| Between |  |  |  |  |  |  |  |  |  |  | |  |  |  |  |  |
| Within |  |  |  |  |  |  |  |  |  |  | |  |  |  |  |  |
| Average | 1.05 (0.89-1.24) | 0.569 | 1.000 | 0.99 (0.82-1.21) | 0.954 | 0.913 | 1.01 (0.82-1.25) | 0.898 | 1.000 | 0.98 (0.87-1.10) | | 0.732 | 1.000 | 0.99 (0.81-1.22) | 0.923 | 0.871 |
| Positive caregiving |  |  |  |  |  |  |  |  |  |  | |  |  |  |  |  |
| Between |  |  |  |  |  |  |  |  |  |  | |  |  |  |  |  |
| Within |  |  |  |  |  |  |  |  |  |  | |  |  |  |  |  |
| Average | 1.00 (0.94-1.06) | 0.955 | 1.000 | 1.00 (0.94-1.07) | 0.951 | 0.913 | 0.98 (0.92-1.05) | 0.612 | 1.000 | 0.99 (0.95-1.03) | | 0.643 | 1.000 | 0.96 (0.89-1.03) | 0.248 | 0.499 |
| Caregiver supervision |  |  |  |  |  |  |  |  |  |  | |  |  |  |  |  |
| Between | 0.94 (0.89-0.99) | 0.025 | 0.210 | 0.94 (0.90-0.99) | 0.023 | 0.066 |  |  |  |  | |  |  | 0.92 (0.86-0.98) | 0.012 | 0.092 |
| Within | 1.02 (0.98-1.07) | 0.323 | 1.000 | 1.07 (1.02-1.13) | 0.012 | 0.066 |  |  |  |  | |  |  | 1.02 (0.97-1.07) | 0.439 | 0.577 |
| Average |  |  |  |  |  |  | 1.00 (0.95-1.04) | 0.821 | 1.000 | 0.98 (0.96-1.00) | | 0.098 | 1.000 |  |  |  |
| Adolescent-caregiver communication |  |  |  |  |  |  |  |  |  |  | |  |  |  |  |  |
| Between |  |  |  |  |  |  |  |  |  |  | |  |  |  |  |  |
| Within |  |  |  |  |  |  |  |  |  |  | |  |  |  |  |  |
| Average | 1.07 (0.97-1.18) | 0.166 | 0.992 | 1.07 (0.97-1.19) | 0.191 | 0.314 | 0.95 (0.85-1.07) | 0.427 | 1.000 | 1.02 (0.95-1.09) | | 0.634 | 1.000 | 1.13 (1.00-1.28) | 0.044 | 0.153 |
| Education enrolment |  |  |  |  |  |  |  |  |  |  | |  |  |  |  |  |
| Average | 0.86 (0.24-3.07) | 0.822 | 1.000 | 2.05 (0.41-10.33) | 0.383 | 0.469 | 1.03 (0.26-4.05) | 0.966 | 1.000 | 0.96 (0.37-2.49) | | 0.934 | 1.000 | 2.29 (0.53-9.81) | 0.266 | 0.499 |
| Days with enough food |  |  |  |  |  |  |  |  |  |  | |  |  |  |  |  |
| Between |  |  |  | 0.68 (0.50-0.94) | 0.020 | 0.066 |  |  |  |  | |  |  |  |  |  |
| Within |  |  |  | 1.19 (0.82-1.75) | 0.360 | 0.469 |  |  |  |  | |  |  |  |  |  |
| Average | 1.08 (0.83-1.40) | 0.554 | 1.000 |  |  |  | 1.02 (0.74-1.39) | 0.918 | 1.000 | 0.95 (0.80-1.14) | | 0.586 | 1.000 | 1.15 (0.83-1.61) | 0.396 | 0.577 |
| HIV status - Living with HIV | 0.66 (0.32-1.34) | 0.246 |  | 1.20 (0.58-2.47) | 0.621 |  | 1.21 (0.54-2.69) | 0.642 |  | 0.67 (0.44-1.04) | | 0.076 |  | 0.26 (0.11-0.64) | 0.003 |  |
| Rural location - Yes | 1.11 (0.51-2.41) | 0.790 |  | 1.28 (0.60-2.76) | 0.523 |  | 0.50 (0.18-1.39) | 0.182 |  | 0.48 (0.28-0.83) | | 0.009 |  | 0.52 (0.19-1.42) | 0.201 |  |
| Informal housing - Yes | 0.63 (0.20-1.95) | 0.420 |  | 1.32 (0.48-3.62) | 0.591 |  | 0.33 (0.06-1.76) | 0.193 |  | 0.96 (0.48-1.91) | | 0.908 |  | 0.15 (0.02-1.07) | 0.059 |  |
| Household size | 0.94 (0.84-1.04) | 0.220 |  | 1.04 (0.94-1.16) | 0.421 |  | 1.02 (0.91-1.15) | 0.732 |  | 1.01 (0.94-1.08) | | 0.798 |  | 1.00 (0.88-1.13) | 0.989 |  |
| Maternal orphan - Yes | 0.84 (0.40-1.78) | 0.644 |  | 0.94 (0.45-1.93) | 0.859 |  | 0.62 (0.26-1.49) | 0.288 |  | 0.52 (0.32-0.85) | | 0.009 |  | 1.05 (0.41-2.69) | 0.918 |  |
| Paternal orphan - Yes | 0.69 (0.37-1.28) | 0.237 |  | 0.86 (0.44-1.70) | 0.669 |  | 0.78 (0.36-1.69) | 0.525 |  | 1.14 (0.72-1.80) | | 0.566 |  | 1.41 (0.64-3.12) | 0.399 |  |
| Age | 2.32 (1.91-2.83) | <.001 |  | 1.48 (1.28-1.71) | <.001 |  | 1.55 (1.32-1.82) | <.001 |  | 1.28 (1.17-1.39) | | <.001 |  | 1.83 (1.49-2.26) | <.001 |  |
| Wave | 0.60 (0.37-0.96) | 0.035 |  | 0.22 (0.11-0.44) | <.001 |  | 0.49 (0.25-0.95) | 0.035 |  | 0.48 (0.33-0.71) | | <.001 |  | 0.69 (0.38-1.25) | 0.216 |  |
| Variance components |  |  |  |  |  |  |  |  |  |  | |  |  |  |  |  |
| Level 2: In level-1 intercept | 4.24 |  |  | 0.27 |  |  | 1.33 |  |  | 0.60 | |  |  | 3.78 |  |  |
| Goodness of fit |  |  |  |  |  |  |  |  |  |  | |  |  |  |  |  |
| AUC | 0.98 |  |  | 0.89 |  |  | 0.95 |  |  | 0.85 | |  |  | 0.99 |  |  |

^a^Wald p-value for significance. A significant p-value indicates that a coefficient is significantly different from 1.00.

Abbreviations: aOR, adjusted odds ratio; CI, confidence interval; signif. significance; equal. Equality; AUC, Area Under the Curve.

**Supplementary Table 13. Adjusted probabilities and probability ratios for HIV risk practices at the mean and maximum of selected protective factors.** Adjusted probabilities were estimated with all covariates at observed values.

|  | Multiple sexual partners | | Transactional sex | | Age-disparate sex | | Condomless sex | | Sex on substances | |
| --- | --- | --- | --- | --- | --- | --- | --- | --- | --- | --- |
|  | Percentage probability (95% CIs) | Probability ratio (95% CIs) | Percentage probability (95% CIs) | Probability ratio (95% CIs) | Percentage probability (95% CIs) | Probability ratio (95% CIs) | Percentage probability (95% CIs) | Probability ratio (95% CIs) | Percentage probability (95% CIs) | Probability ratio (95% CIs) |
| **Girls** |  |  |  |  |  |  |  |  |  |  |
| **Overall** |  |  |  |  |  |  |  |  |  |  |
| Positive caregiving = 19 | 10.07 (8.33; 11.82) | Ref | 4.68 (3.46; 5.90) | Ref | 4.34 (3.02; 5.65) | Ref | 19.17 (17.17; 21.16) | Ref | 3.23 (1.60; 4.86) | Ref |
| Positive caregiving = 24 | 9.51 (7.37; 11.66) | 0.94 (0.84; 1.05) | 3.70 (2.45; 4.95) | 0.79 (0.67; 0.91) | 3.69 (2.28; 5.11) | 0.85 (0.72; 0.99) | 19.24 (16.63; 21.85) | 1.00 (0.93; 1.08) | 3.76 (1.63; 5.88) | 1.16 (0.90; 1.42) |
| **HIV uninfected** |  |  |  |  |  |  |  |  |  |  |
| Positive caregiving = 19 | 9.38 (6.85; 11.90) | Ref | 4.39 (2.74; 6.03) | Ref | 3.95 (2.28; 5.62) | Ref | 22.84 (19.44; 26.24) | Ref | 4.08 (1.68; 6.47) | Ref |
| Positive caregiving = 24 | 8.84 (6.09; 11.59) | 0.94 (0.84; 1.05) | 3.46 (1.93; 4.99) | 0.79 (0.67; 0.91) | 3.36 (1.71; 5.00) | 0.85 (0.71; 0.99) | 22.92 (19.04; 26.80) | 1.00 (0.93; 1.07) | 4.71 (1.74; 7.69) | 1.16 (0.91; 1.40) |
| **Living with HIV** |  |  |  |  |  |  |  |  |  |  |
| Positive caregiving = 19 | 10.44 (8.38; 12.50) | Ref | 4.82 (3.44; 6.20) | Ref | 4.53 (3.03; 6.03) | Ref | 17.19 (14.92; 19.46) | Ref | 2.80 (1.19; 4.41) | Ref |
| Positive caregiving = 24 | 9.86 (7.45; 12.27) | 0.94 (0.84; 1.04) | 3.82 (2.45; 5.18) | 0.79 (0.67; 0.91) | 3.86 (2.29; 5.43) | 0.85 (0.72; 0.99) | 17.25 (14.49; 20.02) | 1.00 (0.93; 1.08) | 3.27 (1.21; 5.32) | 1.17 (0.90; 1.43) |
| **Overall** |  |  |  |  |  |  |  |  |  |  |
| Caregiver supervision = 34 | 7.54 (5.73; 9.35) | Ref | 4.12 (2.92; 5.31) | Ref | 4.11 (2.82; 5.40) | Ref | 17.68 (15.51; 19.85) | Ref | 1.44 (0.28; 2.59) | Ref |
| Caregiver supervision = 40 | 4.46 (2.61; 6.32) | 0.59 (0.46; 0.72) | 3.10 (1.97; 4.22) | 0.75 (0.66; 0.84) | 3.44 (2.12; 4.77) | 0.84 (0.73; 0.95) | 14.15 (11.06; 17.24) | 0.80 (0.69; 0.91) | 0.61 (-0.07; 1.28) | 0.42 (0.26; 0.59) |
| **HIV uninfected** |  |  |  |  |  |  |  |  |  |  |
| Caregiver supervision = 34 | 6.93 (4.53; 9.32) | Ref | 3.85 (2.30; 5.40) | Ref | 3.74 (2.11; 5.37) | Ref | 21.47 (17.85; 25.10) | Ref | 4.08 (1.68; 6.47) | Ref |
| Caregiver supervision = 40 | 4.06 (2.01; 6.11) | 0.59 (0.45; 0.72) | 2.89 (1.54; 4.23) | 0.75 (0.66; 0.84) | 3.12 (1.56; 4.68) | 0.84 (0.72; 0.95) | 17.44 (13.19; 21.68) | 0.81 (0.71; 0.91) | 4.71 (1.74; 7.69) | 0.43 (0.26; 0.59) |
| **Living with HIV** |  |  |  |  |  |  |  |  |  |  |
| Caregiver supervision = 34 | 7.85 (5.80; 9.91) | Ref | 4.25 (2.90; 5.59) | Ref | 4.29 (2.83; 5.76) | Ref | 15.73 (13.34; 18.12) | Ref | 1.22 (0.17; 2.28) | Ref |
| Caregiver supervision = 40 | 4.66 (2.66; 6.66) | 0.59 (0.46; 0.72) | 3.20 (1.96; 4.43) | 0.75 (0.66; 0.84) | 3.60 (2.14; 5.06) | 0.84 (0.73; 0.95) | 12.48 (9.46; 15.51) | 0.79 (0.68; 0.90) | 0.52 (-0.08; 1.11) | 0.42 (0.26; 0.58) |
| **Overall** |  |  |  |  |  |  |  |  |  |  |
| Adol./ caregiver comm. = 34 | 10.20 (8.48; 11.92) | Ref | 5.22 (3.95; 6.48) | Ref | 4.57 (3.26; 5.88) | Ref | 19.02 (17.03; 21.01) | Ref | 2.82 (1.30; 4.34) | Ref |
| Adol./ caregiver comm. = 40 | 9.51 (6.07; 12.94) | 0.93 (0.66; 1.21) | 8.88 (5.02; 12.75) | 1.70 (1.08; 2.32) | 3.73 (1.62; 5.83) | 0.82 (0.45; 1.18) | 16.94 (12.67; 21.22) | 0.89 (0.70; 1.08) | 1.58 (-0.08; 3.25) | 0.56 (0.14; 0.98) |
| **HIV uninfected** |  |  |  |  |  |  |  |  |  |  |
| Adol./ caregiver comm. = 34 | 9.50 (6.97; 12.03) | Ref | 4.91 (3.17; 6.65) | Ref | 4.17 (2.46; 5.88) | Ref | 22.69 (19.28; 26.10) | Ref | 4.08 (1.68; 6.47) | Ref |
| Adol./ caregiver comm. = 40 | 8.84 (4.98; 12.70) | 0.93 (0.65; 1.21) | 8.42 (4.16; 12.68) | 1.72 (1.08; 2.35) | 3.39 (1.13; 5.66) | 0.81 (0.45; 1.18) | 20.37 (14.89; 25.85) | 0.90 (0.72; 1.08) | 4.71 (1.74; 7.69) | 0.57 (0.15; 0.99) |
| **Living with HIV** |  |  |  |  |  |  |  |  |  |  |
| Adol./ caregiver comm. = 34 | 10.56 (8.53; 12.59) | Ref | 5.37 (3.93; 6.82) | Ref | 4.77 (3.27; 6.27) | Ref | 17.05 (14.80; 19.31) | Ref | 2.45 (0.98; 3.92) | Ref |
| Adol./ caregiver comm. = 40 | 9.85 (6.26; 13.45) | 0.93 (0.66; 1.21) | 9.13 (5.11; 13.15) | 1.70 (1.08; 2.32) | 3.89 (1.68; 6.11) | 0.82 (0.46; 1.18) | 15.12 (11.05; 19.19) | 0.89 (0.69; 1.08) | 1.36 (-0.11; 2.83) | 0.56 (0.14; 0.98) |
| **Overall** |  |  |  |  |  |  |  |  |  |  |
| Education enrolment = 0 | 9.91 (7.63; 12.19) | Ref | 4.28 (2.83; 5.73) | Ref | 6.28 (4.16; 8.40) | Ref | 27.04 (20.90; 33.18) | Ref | 3.25 (1.43; 5.07) | Ref |
| Education enrolment = 1 | 10.55 (8.37; 12.73) | 1.06 (0.76; 1.37) | 5.65 (4.05; 7.26) | 1.32 (0.80; 1.84) | 3.10 (1.72; 4.47) | 0.49 (0.26; 0.73) | 15.85 (13.05; 18.65) | 0.59 (0.39; 0.78) | 2.78 (0.98; 4.59) | 0.86 (0.32; 1.39) |
| **HIV uninfected** |  |  |  |  |  |  |  |  |  |  |
| Education enrolment = 0 | 9.23 (6.30; 12.17) | Ref | 4.02 (2.21; 5.82) | Ref | 5.75 (3.14; 8.35) | Ref | 31.96 (23.86; 40.06) | Ref | 4.09 (1.44; 6.74) | Ref |
| Education enrolment = 1 | 9.84 (7.08; 12.61) | 1.07 (0.75; 1.38) | 5.32 (3.38; 7.26) | 1.33 (0.80; 1.85) | 2.81 (1.32; 4.30) | 0.49 (0.26; 0.72) | 19.45 (15.60; 23.30) | 0.61 (0.42; 0.80) | 3.52 (1.07; 5.97) | 0.86 (0.34; 1.38) |
| **Living with HIV** |  |  |  |  |  |  |  |  |  |  |
| Education enrolment = 0 | 10.27 (7.78; 12.75) | Ref | 4.40 (2.86; 5.94) | Ref | 6.57 (4.22; 8.92) | Ref | 24.50 (18.65; 30.35) | Ref | 2.80 (1.08; 4.53) | Ref |
| Education enrolment = 1 | 10.92 (8.40; 13.44) | 1.06 (0.76; 1.36) | 5.81 (4.00; 7.62) | 1.32 (0.80; 1.84) | 3.25 (1.71; 4.79) | 0.50 (0.26; 0.73) | 13.97 (10.96; 16.97) | 0.57 (0.37; 0.77) | 2.39 (0.64; 4.14) | 0.85 (0.31; 1.40) |
| **Overall** |  |  |  |  |  |  |  |  |  |  |
| No. of days with enough food = 6 | 10.52 (8.75; 12.29) | Ref | 5.07 (3.78; 6.36) | Ref | 4.51 (3.21; 5.80) | Ref | 19.74 (17.58; 21.90) | Ref | 3.10 (1.52; 4.69) | Ref |
| No. of days with enough food = 7 | 9.32 (7.54; 11.10) | 0.89 (0.81; 0.97) | 4.16 (2.92; 5.40) | 0.82 (0.72; 0.92) | 5.02 (3.54; 6.51) | 1.11 (0.95; 1.28) | 18.04 (15.67; 20.42) | 0.91 (0.81; 1.02) | 2.88 (1.29; 4.46) | 0.93 (0.72; 1.13) |
| **HIV uninfected** |  |  |  |  |  |  |  |  |  |  |
| No. of days with enough food = 6 | 9.80 (7.20; 12.41) | Ref | 4.76 (3.00; 6.51) | Ref | 4.11 (2.44; 5.79) | Ref | 23.53 (19.89; 27.17) | Ref | 3.92 (1.59; 6.25) | Ref |
| No. of days with enough food = 7 | 8.66 (6.20; 11.11) | 0.88 (0.80; 0.97) | 3.89 (2.33; 5.46) | 0.82 (0.72; 0.91) | 4.60 (2.73; 6.47) | 1.12 (0.95; 1.28) | 21.64 (18.05; 25.22) | 0.92 (0.82; 1.02) | 3.64 (1.35; 5.93) | 0.93 (0.73; 1.13) |
| **Living with HIV** |  |  |  |  |  |  |  |  |  |  |
| No. of days with enough food = 6 | 10.91 (8.81; 13.00) | Ref | 5.23 (3.76; 6.70) | Ref | 4.70 (3.22; 6.18) | Ref | 17.70 (15.30; 20.10) | Ref | 2.68 (1.13; 4.23) | Ref |
| No. of days with enough food = 7 | 9.67 (7.58; 11.76) | 0.89 (0.81; 0.97) | 4.29 (2.89; 5.69) | 0.82 (0.73; 0.92) | 5.24 (3.54; 6.93) | 1.11 (0.95; 1.27) | 16.11 (13.50; 18.71) | 0.91 (0.80; 1.02) | 2.48 (0.93; 4.02) | 0.92 (0.72; 1.13) |
| **Boys** |  |  |  |  |  |  |  |  |  |  |
| **Overall** |  |  |  |  |  |  |  |  |  |  |
| Positive caregiving = 19 | 12.34 (10.53; 14.14) | Ref | 3.07 (2.27; 3.87) | Ref | 1.82 (0.69; 2.95) | Ref | 9.26 (7.42; 11.10) | Ref | 5.41 (3.38; 7.43) | Ref |
| Positive caregiving = 24 | 12.40 (10.05; 14.75) | 1.00 (0.90; 1.11) | 2.37 (1.37; 3.37) | 0.77 (0.59; 0.96) | 1.87 (0.54; 3.20) | 1.03 (0.73; 1.32) | 11.66 (9.01; 14.32) | 1.26 (1.08; 1.43) | 6.33 (3.56; 9.10) | 1.17 (0.93; 1.41) |
| **HIV uninfected** |  |  |  |  |  |  |  |  |  |  |
| Positive caregiving = 19 | 14.82 (11.63; 18.02) | Ref | 2.74 (1.52; 3.95) | Ref | 1.59 (0.36; 2.82) | Ref | 12.21 (8.96; 15.46) | Ref | 8.59 (4.98; 12.21) | Ref |
| Positive caregiving = 24 | 14.89 (11.30; 18.49) | 1.00 (0.91; 1.10) | 2.11 (0.95; 3.27) | 0.77 (0.59; 0.96) | 1.64 (0.26; 3.01) | 1.03 (0.73; 1.32) | 15.25 (10.99; 19.51) | 1.25 (1.08; 1.42) | 9.96 (5.42; 14.51) | 1.16 (0.94; 1.38) |
| **Living with HIV** |  |  |  |  |  |  |  |  |  |  |
| Positive caregiving = 19 | 11.00 (8.87; 13.13) | Ref | 3.27 (2.22; 4.31) | Ref | 1.98 (0.66; 3.29) | Ref | 7.76 (5.83; 9.69) | Ref | 3.57 (1.59; 5.56) | Ref |
| Positive caregiving = 24 | 11.05 (8.46; 13.65) | 1.01 (0.90; 1.11) | 2.53 (1.34; 3.72) | 0.77 (0.59; 0.96) | 2.03 (0.50; 3.56) | 1.03 (0.74; 1.32) | 9.91 (7.20; 12.62) | 1.28 (1.09; 1.47) | 4.29 (1.66; 6.91) | 1.20 (0.91; 1.49) |
| **Overall** |  |  |  |  |  |  |  |  |  |  |
| Caregiver supervision = 33 | 10.82 (8.89; 12.76) | Ref | 2.59 (1.82; 3.36) | Ref | 1.43 (0.41; 2.44) | Ref | 9.03 (7.19; 10.88) | Ref | 3.74 (1.87; 5.61) | Ref |
| Caregiver supervision = 40 | 7.93 (5.88; 9.98) | 0.73 (0.64; 0.82) | 1.63 (0.90; 2.36) | 0.63 (0.50; 0.76) | 0.96 (0.13; 1.79) | 0.67 (0.49; 0.85) | 8.38 (6.23; 10.54) | 0.93 (0.81; 1.05) | 2.30 (0.71; 3.89) | 0.61 (0.45; 0.78) |
| **HIV uninfected** |  |  |  |  |  |  |  |  |  |  |
| Caregiver supervision = 33 | 13.32 (9.95; 16.69) | Ref | 2.29 (1.15; 3.42) | Ref | 1.23 (0.16; 2.31) | Ref | 11.96 (8.69; 15.23) | Ref | 6.37 (2.87; 9.87) | Ref |
| Caregiver supervision = 40 | 9.88 (6.74; 13.02) | 0.74 (0.66; 0.83) | 1.43 (0.56; 2.31) | 0.63 (0.50; 0.76) | 0.83 (-0.01; 1.67) | 0.67 (0.49; 0.85) | 11.12 (7.60; 14.65) | 0.93 (0.82; 1.04) | 3.99 (1.09; 6.88) | 0.63 (0.46; 0.80) |
| **Living with HIV** |  |  |  |  |  |  |  |  |  |  |
| Caregiver supervision = 33 | 9.45 (7.26; 11.65) | Ref | 2.76 (1.80; 3.71) | Ref | 1.55 (0.39; 2.70) | Ref | 7.55 (5.65; 9.46) | Ref | 2.29 (0.70; 3.87) | Ref |
| Caregiver supervision = 40 | 6.80 (4.67; 8.92) | 0.72 (0.63; 0.81) | 1.74 (0.90; 2.57) | 0.63 (0.50; 0.76) | 1.04 (0.11; 1.97) | 0.67 (0.50; 0.85) | 6.98 (4.90; 9.06) | 0.92 (0.80; 1.05) | 1.34 (0.18; 2.51) | 0.59 (0.42; 0.76) |
| **Overall** |  |  |  |  |  |  |  |  |  |  |
| No. of days with enough food = 6 | 12.32 (10.54; 14.11) | Ref | 3.25 (2.47; 4.03) | Ref | 1.81 (0.69; 2.92) | Ref | 9.17 (7.34; 11.00) | Ref | 5.13 (3.17; 7.08) | Ref |
| No. of days with enough food = 7 | 12.39 (10.52; 14.25) | 1.00 (0.96; 1.05) | 2.97 (2.17; 3.76) | 0.91 (0.84; 0.98) | 1.85 (0.69; 3.01) | 1.02 (0.89; 1.15) | 9.14 (7.22; 11.05) | 1.00 (0.94; 1.05) | 5.20 (3.16; 7.23) | 1.01 (0.92; 1.11) |
| **HIV uninfected** |  |  |  |  |  |  |  |  |  |  |
| No. of days with enough food = 6 | 14.81 (11.62; 18.00) | Ref | 2.89 (1.62; 4.17) | Ref | 1.58 (0.36; 2.80) | Ref | 12.10 (8.85; 15.35) | Ref | 8.20 (4.63; 11.77) | Ref |
| No. of days with enough food = 7 | 14.88 (11.61; 18.14) | 1.00 (0.96; 1.05) | 2.64 (1.41; 3.87) | 0.91 (0.84; 0.98) | 1.62 (0.35; 2.88) | 1.02 (0.89; 1.15) | 12.06 (8.74; 15.38) | 1.00 (0.94; 1.05) | 8.31 (4.62; 11.99) | 1.01 (0.93; 1.10) |
| **Living with HIV** |  |  |  |  |  |  |  |  |  |  |
| No. of days with enough food = 6 | 10.98 (8.88; 13.09) | Ref | 3.45 (2.43; 4.46) | Ref | 1.96 (0.67; 3.26) | Ref | 7.67 (5.76; 9.59) | Ref | 3.34 (1.46; 5.22) | Ref |
| No. of days with enough food = 7 | 11.04 (8.88; 13.20) | 1.01 (0.96; 1.05) | 3.15 (2.15; 4.15) | 0.91 (0.84; 0.98) | 2.01 (0.67; 3.34) | 1.02 (0.89; 1.15) | 7.64 (5.68; 9.61) | 1.00 (0.94; 1.06) | 3.39 (1.46; 5.32) | 1.02 (0.91; 1.12) |

Multivariable models were adjusted for participant age, HIV status, rural/urban location, informal housing, household size, maternal orphanhood, paternal orphanhood, and study wave. ^a^Sex on substances was only measured at wave two and wave three.

Abbreviations: CI, confidence interval; PPT, percentage point.
